# Systematic analysis of BRAF^V^^600E^ melanomas reveals a role for JNK/c-Jun pathway in adaptive resistance to drug-induced apoptosis

**DOI:** 10.15252/msb.20145877

**Published:** 2015-03-26

**Authors:** Mohammad Fallahi-Sichani, Nathan J Moerke, Mario Niepel, Tinghu Zhang, Nathanael S Gray, Peter K Sorger

**Affiliations:** 1 HMS LINCS Center, Department of Systems Biology, Harvard Medical SchoolBoston, MA, USA; 2 Department of Cancer Biology, Dana-Farber Cancer Institute, Harvard Medical SchoolBoston, MA, USA; 3 Department of Biological Chemistry and Molecular Pharmacology, Harvard Medical SchoolBoston, MA, USA

**Keywords:** adaptive responses, BRAF^V^^600E^ melanomas, cell-to-cell variability, RAF and MEK inhibitors, submaximal drug effect

## Abstract

Drugs that inhibit RAF/MEK signaling, such as vemurafenib, elicit profound but often temporary anti-tumor responses in patients with BRAF^V^^600E^ melanoma. Adaptive responses to RAF/MEK inhibition occur on a timescale of hours to days, involve homeostatic responses that reactivate MAP kinase signaling and compensatory mitogenic pathways, and attenuate the anti-tumor effects of RAF/MEK inhibitors. We profile adaptive responses across a panel of melanoma cell lines using multiplex biochemical measurement, single-cell assays, and statistical modeling and show that adaptation involves at least six signaling cascades that act to reduce drug potency (IC_50_) and maximal effect (i.e., *E*_max_ ≪ 1). Among these cascades, we identify a role for JNK/c-Jun signaling in vemurafenib adaptation and show that RAF and JNK inhibitors synergize in cell killing. This arises because JNK inhibition prevents a subset of cells in a cycling population from becoming quiescent upon vemurafenib treatment, thereby reducing drug *E*_max_. Our findings demonstrate the breadth and diversity of adaptive responses to RAF/MEK inhibition and a means to identify which steps in a signaling cascade are most predictive of phenotypic response.

## Introduction

Activation of BRAF via a V600E (or V600D) mutation is the most prevalent genetic change in human melanoma, found in at least 50% of tumors. The BRAF^V600E^ oncoprotein constitutively activates pro-mitogenic RAF/MEK/ERK signaling (Davies *et al*, 2002; Fecher *et al*, 2008), and therapy with RAF inhibitors such as vemurafenib (Zelboraf®; PLX4032) causes tumor regression in many patients (Bollag *et al*, 2010; Chapman *et al*, 2011; Flaherty *et al*, 2012; Sosman *et al*, 2012). However, the duration of response is variable and relapse to lethal drug-resistant disease is common (Flaherty *et al*, 2010). Resistance usually involves the activation of pro-growth/survival mechanisms that increase BRAF^V600E^ activity (Shi *et al*, 2012) or bypass the need for it altogether. Many mutations involved in acquired resistance have been identified, including NRAS^Q61K^, MEK1^F129L^, MEK2^Q60P^, or AKT1^Q79K^ (Nazarian *et al*, 2010; Wang *et al*, 2011; Shi *et al*, 2014a; Wagle *et al*, 2014), and aberrant splicing of BRAF^V600E^ (Poulikakos *et al*, 2011). Resistance is also associated with elevated IGF1 receptor/PI3K signaling (Villanueva *et al*, 2010), COT overexpression (Johannessen *et al*, 2010), and PDGFRβ up-regulation (Nazarian *et al*, 2010).

Insensitivity to RAF inhibition in BRAF^V600E^ melanomas frequently arises from adaptive responses that reactivate ERK (Lito *et al*, 2012) or up-regulate other pro-growth pathways such as the PI3K/AKT cascade (Shi *et al*, 2014a; Sun *et al*, 2014). Adaptive responses are thought to reflect feedback mechanisms involved in signaling homeostasis (O'Reilly *et al*, 2006; Carver *et al*, 2011; Chandarlapaty *et al*, 2011). Changes consistent with adaptation have been observed not only in cell lines but also in clinical biopsies (Chandarlapaty, 2012; Duncan *et al*, 2012; Lito *et al*, 2012; Muranen *et al*, 2012; Shi *et al*, 2014a; Solit & Rosen, 2014), and it is thought that the resulting “partial” response to RAF inhibition increases the probability that genetic changes leading to acquired resistance will arise (Lito *et al*, 2013; Shi *et al*, 2014a,b).

The study of adaptive responses provides a window into the complex and still poorly understood networks involved in feedback regulation of mitogenic signaling, and preventing adaptation is likely to be key to durable therapy. However, systematic data comparing BRAF^V600E^ lines are generally lacking, and it is not known whether adaptation to different MEK and RAF inhibitors is fundamentally similar or whether multiple adaptive mechanisms are active in a single cell line or tumor. It is also unclear whether the key difference between sensitive and resistant cells involves drug potency (difference in IC_50_) or the fraction of cells that are responsive (*E*_max_). Variation in *E*_max_ and in the slope of the dose–response curve can play a significant role in limiting the efficacy of anti-cancer drugs (Fallahi-Sichani *et al*, 2013).

In this study, we profile the responses of human BRAF^V600E/D^ melanoma lines to RAF and MEK inhibitors to: (i) characterize variability in adaptation with time, dose, and genotype, (ii) discover new adaptive mechanisms, and (iii) compare phenotypes of adaptation at single-cell and population levels. We apply a three-step approach involving measurement of multiple signaling proteins across dose and time combined with population average and single-cell measurement of cell state and phenotype followed by statistical modeling. Our data comprise reverse-phase protein array (RPPA) measurement of 17 signaling proteins and 4 cell state markers as well as single-cell assays of apoptosis and cell viability in 10 lines exposed to five drugs for 1–72 h at 7 doses spanning IC_50_. Statistical modeling using partial least squares regression (PLSR) was then used to determine which of the ∽2 × 10^5^ data points were predictive of drug-induced changes in viability and apoptosis; follow-on experiments tested these predictions. We find that adaptive responses to RAF or MEK inhibition are diverse with time and genotype and involve six or more signaling cascades (e.g., AKT/mTOR, NF-κB, and AMPK), but are similar for different drugs, excluding known differences in mechanism of action and polypharmacology. We find that the JNK/c-Jun pathway was initially down-regulated by RAF/MEK inhibitors in all cell lines but in half of the lines, it was then significantly up-regulated. In 4 of 10 lines, JNK/c-Jun up-regulation caused a subset of cells to become quiescent and apoptosis-resistant. The addition of an irreversible JNK inhibitor (Zhang *et al*, 2012) synergized with vemurafenib in cell killing, primarily by increasing *E*_max_. Thus, co-treatment of some melanomas with RAF and JNK inhibitors may have clinical potential, analogous to RAF and PI3K/AKT inhibitors (Jang & Atkins, 2014).

## Results

### Data-driven modeling of adaptive responses to RAF and MEK inhibition

**Figure 1:**
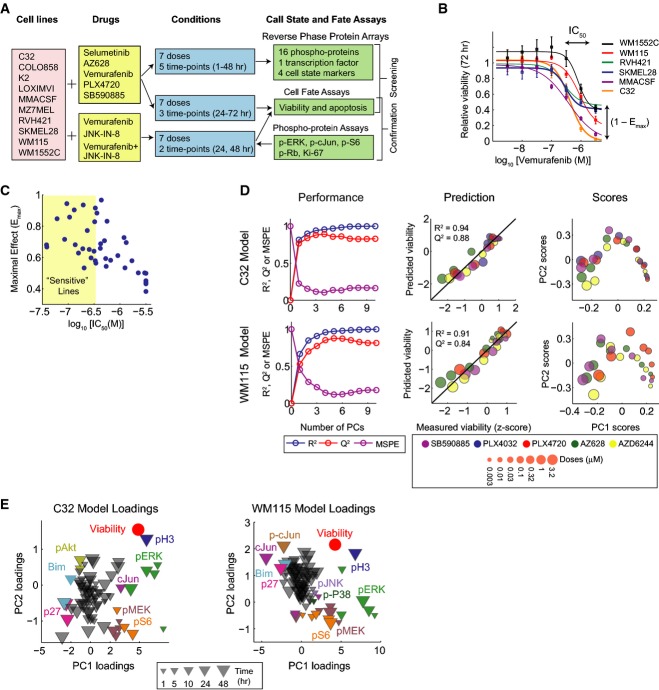
Data-driven modeling reveals signaling correlates of BRAF-mutant melanoma cellular response to RAF/MEK inhibition

To profile adaptive responses in melanoma, we applied single-cell phenotypic and multiplex biochemical assays to nine BRAF^V600E^ and one BRAF^V600D^ lines exposed to four RAF inhibitors and one MEK inhibitor at multiple doses and times (Fig1A). Seven of the tested cell lines have been genetically characterized through the Cancer Genome Project (Garnett *et al*, 2012) (Supplementary Dataset S1). Cell viability and induction of apoptosis were scored using automated fluorescence microscopy and two dyes: DEVD-NucView488 for effector caspases (Tang *et al*, 2013) and Hoechst 33342 for nuclei (Supplementary Fig S1A). Phenotypic assays were performed 24, 48, and 72 h following exposure to four RAF inhibitors with differential selectivity for BRAF^V600E^, wild-type BRAF and CRAF, including AZ628, vemurafenib, PLX4720 (a structural analogue of vemurafenib), and SB590885 as well as the phase III MEK inhibitor selumetinib (AZD6244). Variability was observed in IC_50_ and *E*_max_ with drug and cell type (Fallahi-Sichani *et al*, 2013), implying that fractional cell killing is common even among more sensitive cell lines (Fig1B and C and Supplementary Fig S1B).

Overview of the systematic measurements using single-cell phenotypic and multiplex biochemical assays to profile signaling biochemistry and cell state in 10 BRAF^V^^600E/D^ melanoma cell lines exposed to four RAF inhibitors and one MEK inhibitor at multiple doses and times. Multiplex single-cell immunofluorescence assays were used in the follow-up experiments on selected cell lines for treatments with vemurafenib, JNK-IN-8, and their combination.

Dose–response curves representing 72-h viability measurements for six selected BRAF^V^^600E^ melanoma cell lines after treatment with vemurafenib. Ranges of estimated IC_50_ and *E*_max_ for the selected lines are shown. Data are represented as mean ± SD.

Pairwise distribution and correlation of estimated IC_50_ and *E*_max_ values for responses (72-h viability) of 8 cell lines to four RAF inhibitors and one MEK inhibitor. Each dot represents one drug/cell line combination. Sensitive cell lines are arbitrarily defined based on their IC_50_s (the most widely used metric for evaluation of drug response; log_10_[IC_50_] < −6.5).

Cell line-specific model calibration for two selected cell lines C32 and WM115. Left: *R*^2^, *Q*^2^, and MSPE for C32 and WM115 models built with increasing numbers of PLSR components. Middle: Relative viability as measured experimentally (*z*-score-scaled) or as predicted by the three-component PLSR models using tenfold cross-validation. *R*^2^ reports model fit, and *Q*^2^ reports model prediction accuracy. Right: PLSR score plots for the first two components of C32 and WM115 models. Shown PLSR models were developed using RPPA data from Supplementary Dataset S3.

PLSR loading plots for the first two components of C32 and WM115 models.

Source data are available online for this figure.

Signaling proteins were assayed at seven drug doses between 3.2 nM and 3.2 μM and five time points between 1 and 48 h by RPPA (Sevecka *et al*, 2011) using antibodies with good coverage of cell growth, apoptosis, stress response, and energy homeostasis pathways as well as 4 cell state markers (see Materials and Methods for details). Four biological replicates yielded a dataset of ∽180,000 measurements in which multiple signaling proteins were up- or down-regulated depending on cell line and dose (Supplementary Datasets S2 and S3, http://lincs.hms.harvard.edu/db/datasets/20218/). Time was an important variable in these data since pathways down-regulated immediately after drug exposure were frequently up-regulated at later times. To compute the relative importance of each signaling protein for phenotypic responses, we used PLSR (Geladi & Kowalski, 1986; Janes & Yaffe, 2006), generating one model for each cell line. For simplicity, a single “viability” response variable was created by subtracting the number of apoptotic cells from total cell number followed by normalization to a DMSO-treated control and averaging 48- and 72-h data (Supplementary Dataset S4, http://lincs.hms.harvard.edu/db/datasets/20217/).

Input vectors were constructed by normalizing RPPA data, treating dose, time, and drug as separate observations, and then reduced by PLSR, so that each PLSR component (PC) maximally captured variance in the data left unexplained by preceding components. The process was iterated until additional PCs did not improve the prediction relative to experimental noise. Models were evaluated by computing the percent of variance predicted using tenfold cross-validation (*Q*^2^) and the mean squared prediction error (MSPE; Fig1D; left panels) (see Materials and Methods for details). PLSR models proved remarkably accurate with *Q*^2^ = 0.84 ± 0.13 (for PC1-3) and MSPE = 15–20%, close to the estimated error in the array data. As described below, we also performed independent experiments to verify key predictions.

Partial least squares regression models are most useful if they substantially reduce the complexity of the data as evaluated by the fraction of variance in output variables (phenotypes in this case) captured by a small number of PCs (as assessed by *R*^2^). For example, the C32 PLSR model captured 94% of variance in three PCs and the WM115 model captured 91% (Fig1D; middle panels) implying that PLSR could provide meaningful insight into the connection between drug-induced signaling and phenotype. This was important because there is no *a priori* reason to believe that we had selected the right proteins and time points to measure. The high values obtained for *R*^2^ and *Q*^2^ demonstrate that RPPA measurements successfully captured the fraction of variation in signaling across lines, drugs, and times that is consequential for drug response. Data on additional proteins will be needed, of course, to fully map networks involved in adaptation at a molecular level.

In PLSR models, score vectors corresponding to variation in drug doses projected negatively along PC1, visible in Fig1D (right panels) as a left to right progression from high doses (large markers) to low doses (small markers). Data from different drugs projected largely along PC2, as evidenced by changes in color (clearest in the case of the WM115 model). Projecting loading vectors into PLSR component space (Fig1E) revealed the protein changes associated with phenotypic responses. For example, in PC2 which captures drug-dependent differences, RAF but not MEK inhibitors reduced pMEK^(Ser217/221)^ levels at early time points. This arises because kinase inhibition changes the modification state of the substrate rather than the kinase; in some cell lines, pMEK^(Ser217/221)^ levels increased upon selumetinib exposure, a manifestation of feedback regulation. Phospho-p38^(Thr180/Tyr182)^ levels also fell within 1 h of exposure to vemurafenib, PLX4720, or AZ628; KINOMEscan binding data for these drugs (Vin *et al*, 2013) suggest that they have off-target activity on regulators of p38 such as ZAK kinases. We observed that co-treatment of LOXIMVI cells with SB202190, a specific inhibitor of p38 kinase, diminished the effectiveness of vemurafenib and AZ628 (Supplementary Fig S1C–E). Thus, off-target effects of RAF inhibitors on the p38 pathways are likely to be undesirable from a therapeutic perspective. Overall, we conclude that adaptive responses to RAF and MEK inhibitors are diverse across cell lines but similar for different drugs, excluding known off-target effects and differences in potency.

Partial least squares regression loadings were generally interpretable in molecular terms. For example, increasing drug dose correlated with lower pERK^(Thr202/Tyr204)^ levels and up-regulation of PI3K/AKT signaling (Shi *et al*, 2014a). The correlation structure in the loadings was also interpretable: PC1 showed a strong positive correlation between growth/survival signals down-regulated by drug (Fig1E; pERK^(Thr202/Tyr204)^, pS6^(Ser235/236)^ in green and orange) and markers of mitotic state (phospho-histone H3; pH3—dark blue) and a negative correlation with the quiescence marker p27 (dark pink) and the apoptosis inducer Bim (a Bcl2 family member—light blue). We observed equally significant changes in other signaling pathways, including those involved in stress/cytokine responses (JNK, p38), energy homeostasis (AMPK), and cytokine signal transduction (NF-κB). These data are consistent with a complex adaptive response involving multiple signal transduction cascades in different combinations in each cell line.

### c-Jun activity up-regulation by RAF inhibitors causes resistance to apoptosis

**Figure 2:**
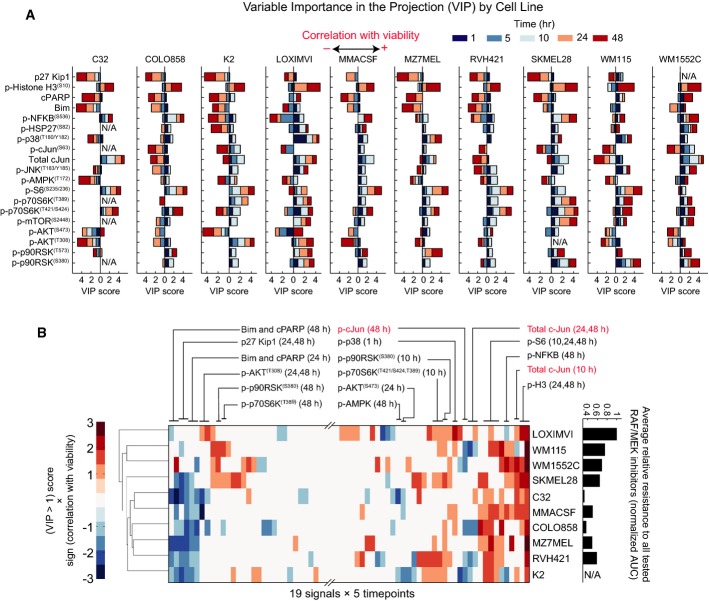
Variability in the magnitude, direction, and timing of signaling changes in response to RAF/MEK inhibition

To identify adaptive changes that are predictive of drug response, we calculated the “variable importance in the projection” (VIP) for each of ten PLSR models. VIP scores report the sum (over all model dimensions) of each variable *x* (an RPPA assay at a specific time point), weighted by the change in response (cell viability) explained by the same variable *x* (see Materials and Methods for mathematical details) (Wold, 1994; Janes *et al*, 2008). We generated a VIP score for each three-way cell line/signal/time point combination. Analyzing these scores (Fig2) shows which biochemical changes are common or different across different cell lines and to what extent they occur on similar or different timescales. We found that phospho-protein signals varied markedly with respect to direction (up or down) and timing (early or late; Fig2A). Some cell lines up-regulated a particular pathway, whereas others down-regulated it (e.g., NF-κB and c-Jun). Because VIP scores are always positive, a minus sign was assigned to scores anti-correlated with viability and a significance threshold of |VIP| >1 was then imposed. Unsupervised clustering of positive and negative VIP scores for each model made it possible to visualize changes in signaling associated with sensitivity to RAF/MEK inhibitors (Fig2B; pMEK and pERK scores are not shown because they are a direct consequence of drug action).

PLSR-derived variable importance in the projection (VIP) scores predicting viability for each of the ten studied cell lines. VIP scores are shown for each cell line-specific model, each signal and measurement time point. The direction of the bars (left or right) shows whether the changes in signal correlated negatively or positively with relative viability. VIP scores of larger than one indicate important variables (signals and time points) that predict the responses (viability).

Unsupervised clustering of melanoma cell lines based on the VIP scores >1 from each individual cell line model (excluding pMEK and pERK). Prior to clustering, VIP scores of between 0 and 1 were set to zero and a minus sign was added to VIP scores associated with signals that negatively correlated with viability. Average relative resistance of the cell lines to the five tested RAF/MEK inhibitors (on the right) is computed based on area under the time–dose–response curve plotted for non-apoptotic viability measured by single-cell imaging across seven doses and two time points (48 and 72 h) following treatment. The data for the cell line K2 are not shown because clumping made it difficult to score single-cell phenotypes in this cell line after 72 h.

Source data are available online for this figure.

**Figure 3:**
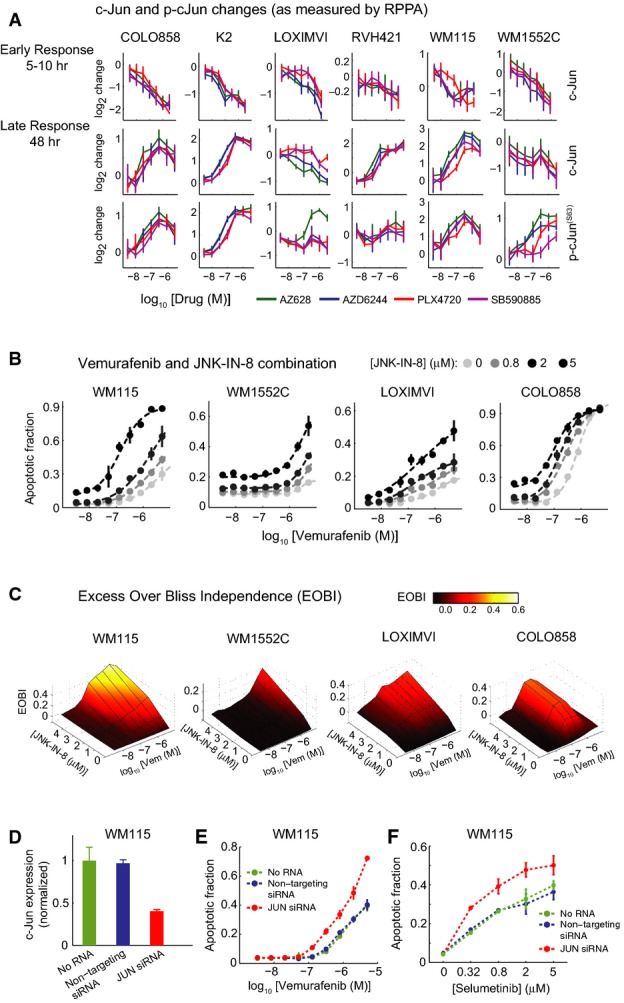
c-Jun activity up-regulation by RAF inhibitors causes resistance to apoptosis

We focused on VIP scores that were significant in multiple cell lines. These include measures of PI3K/AKT signaling, which is known to be activated during vemurafenib adaptation, as well as the levels of c-Jun and/or p-cJun^(Ser63)^. Both rose in multiple cell lines 24–48 h after exposure to RAF and MEK inhibitors. This was unexpected since previous studies report that MEK/ERK signaling regulates c-Jun expression in BRAF^V600E^ melanomas and, thus, that RAF and MEK inhibitors should down-regulate c-Jun (Lopez-Bergami *et al*, 2007). This is what we observed 5–10 h after drug exposure in all lines, but in 6 of 10 lines, p-cJun levels then increased by 24–48 h (and in 4 of these, total levels of c-Jun also increased) (Fig3A). In some cell lines (e.g., WM115) RPPA data demonstrated c-Jun up-regulation as early as *t* = 10 h, whereas in others (e.g., K2) it occurred only after 48 h; these data were confirmed for PLX4720 using single-cell assays (Supplementary Fig S2A and B). Among the cell lines that up-regulate p-cJun, there was a significant correlation between the degree of MEK or RAF inhibition (as assayed by pERK levels) and the magnitude of p-cJun up-regulation. For example, AZ628 induced p-cJun up-regulation at lower doses as compared with vemurafenib and was also a more potent inhibitor of the MAPK pathway. This suggests a previously undescribed role for c-Jun in adaptation of melanoma cells to RAF and MEK inhibition; we therefore focused on JNK/c-Jun in follow-up studies.

A c-Jun and p-c-Jun^(Ser63)^ changes as measured by RPPA in six melanoma cell lines in response to different doses of RAF and MEK inhibitors for 10 h (or 5 h in the case of WM115 cell line) and 48 h.

B, C Synergistic apoptosis induction in four cell lines (WM115, WM1552C, LOXIMVI, and COLO858) treated for 72 h with combinations of vemurafenib and JNK-IN-8. (B) Dose–response profiles for apoptosis induction with vemurafenib and JNK-IN-8 combination. (C) Excess over the predicted Bliss independence (EOBI) calculated for different combined doses of vemurafenib and JNK-IN-8.

D c-Jun expression in WM115 cells transfected with JUN siRNA relative to no RNA and non-targeting controls quantified in triplicate 48 h after transfection.

E, F Apoptosis in WM115 cells with or without 48 h JUN knockdown after 96-h treatment with increasing doses of vemurafenib (E) and selumetinib (F).

Data information: Data are presented as mean ± SD.Source data are available online for this figure.

To determine whether the up-regulation of c-Jun impacted vemurafenib-mediated cell killing, we co-treated cells with JNK-IN-8, a kinase inhibitor that is specific for JNK relative to other MAP family kinases (Zhang *et al*, 2012). In melanoma cells, JNK-IN-8 caused dose-dependent inhibition of c-Jun S63/S73 phosphorylation, a modification required for transcriptional activity, but did not measurably alter the levels of pERK, pAKT^(Ser473)^, pSTAT3^(Tyr705)^, the p38/MK2 substrate pHSP27^(Ser82)^ or the nuclear translocation of NF-κB (Supplementary Fig S2C–I). These modification states report on potential off-target activities of JNK-IN-8, and the absence of significant changes suggests that the drug acted in a JNK-specific manner in melanoma cells at the doses we used. Exposure of cells to JNK-IN-8 alone reduced p-cJun^(Ser73)^ to background levels but induced little if any apoptosis (Fig3B). When cells were co-treated with vemurafenib and JNK-IN-8, p-cJun^(Ser73)^ was also reduced to background levels (Supplementary Fig S2J) and the level of apoptosis was increased, particularly in the three cell lines that were among the most vemurafenib-resistant (WM115, WM1552C, and LOXIMVI) as well as in relatively sensitive COLO858 cells (Fig3B). The EC_50_ for vemurafenib-mediated apoptosis fell by as much as 20-fold, and *E*_max_ increased by up to fivefold. Apoptosis was not increased by JNK-IN-8 in RVH421 cells, but vemurafenib induced little apoptosis in these cells even at the highest doses (Supplementary Fig S2K); in K2 cells, the data were ambiguous because clumping made it difficult to score single-cell phenotypes.

Bliss independence (Keith *et al*, 2005) is a better metric of drug interaction than changes in IC_50_ values, and we observed that JNK-IN-8 was synergistic with vemurafenib in all four lines tested based on excess over Bliss independence (EOBI; Fig3C). Significant but quantitatively modest increases in apoptosis were also observed upon co-treatment of cells with vemurafenib and SP600125, a structurally distinct but less selective JNK inhibitor (Supplementary Fig S2L). Moreover, depletion of *JUN* using siRNA significantly potentiated apoptosis induced by vemurafenib or selumetinib in WM115 and WM1552C lines (Fig3D–F and Supplementary Fig S2M–O) as compared to cells transfected with control siRNA. For 25 BRAF^V600E^ melanoma lines in the Cancer Cell Line Encyclopedia (Barretina *et al*, 2012), we also observed a statistically significant correlation between *JUN* expression levels and PLX4720 sensitivity (Spearman's ρ = 0.47, *P *=* *0.02; see below; Supplementary Fig S2P). We conclude that the combination of RAF and JNK inhibition (or *JUN* depletion) increases apoptosis in some vemurafenib-resistant cell lines to a level normally observed in sensitive cells, implying that the up-regulation of JNK/c-Jun in melanoma cells following vemurafenib exposure decreases cell killing and that the combination of RAF and JNK inhibitors may have therapeutic potential.

### A network perspective on adaptive responses

**Figure 4:**
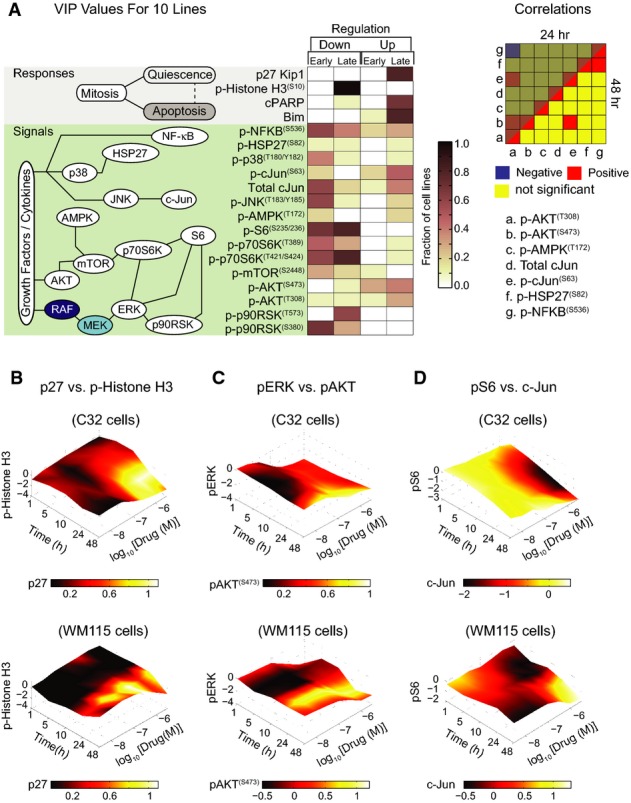
A network perspective on adaptive responses

Mapping VIP values onto a schematic of immediate-early signaling (Fig4A) reveals the diversity of adaptive responses to RAF and MEK inhibition with respect to magnitude and timing (Fig4A). In nearly all cell lines, the quiescence marker p27 and apoptosis markers cPARP and Bim were up-regulated and mitotic marker pH3 down-regulated 24–48 h after drug exposure. Whereas exposure of C32 cells to PLX4720 led to early and significant increase in p27 and decrease in pH3, responses occurred later and were smaller in WM115 cells. These changes are depicted in Fig4B–D with levels of one protein mapped onto a red to yellow color scale and the other protein onto the vertical axis; the *x*–*y* axes represent time and dose. The induction of AKT signaling is among the best described and most common adaptations to RAF inhibition (Shi *et al*, 2014a). In our data, pAKT^(Ser473)^ and/or pAKT^(Thr308)^ rose in both vemurafenib-sensitive lines such as C32 and vemurafenib-resistant lines such as WM115 (Fig4C). Proteins that integrate ERK and AKT signaling such as p-p70S6K^(Thr421/Ser424)^ kinase and pS6^(Ser235/236)^, known to be important in melanoma (Corcoran *et al*, 2013), were down-regulated soon after exposure of some cell lines to drug and only much later in others (compare pS6 levels in C32 and WM115 cells exposed to PLX4720; Fig4D). Finally, pNF-κB^(Ser536)^, pJNK^(Thr183/Tyr185)^, c-Jun, and p-cJun^(Ser63)^ were down-regulated soon (1–10 h) after drug exposure and then up-regulated subsequently (24–48 h) in some lines (Fig4A and D) but not in others. In most cases, we were unable to identify statistically significant correlations between different pathways across cell lines (Fig4A; right panel), implying independence of adaptive mechanisms.

A Left: A consolidated overview of PLSR-derived variable importance in the projection (VIP) scores mapped onto a simple schematic of immediate-early signaling, indicating the fraction of 10 studied cell lines in which early (1–10 h) or late (24–48 h) up- or down-regulation of each of the cell state markers and phospho-proteins is predictive of response to RAF/MEK inhibitors (72-h viability). Up-regulation or down-regulation of signals with VIP score >1 is shown. Right: Correlation between up- and down-regulation of selected pathways in response to RAF/MEK inhibition across the 10 studied cell lines. Correlations between signaling changes in response to RAF/MEK inhibition were evaluated based on pairwise Spearman's correlation between VIP scores for selected phospho-proteins at 24 and 48 h post-treatment across the 10 studied cell lines. *P*-values <0.05 were considered significant.

B-D Time–dose–response plots indicating changes in RPPA measurements for six selected signals, p27 versus p-histone H3 (B), pERK^(Thr202/Tyr204)^ versus pAKT^(Ser473)^ (C), and pS6^(Ser235/236)^ versus total c-Jun levels (D), for two selected cell lines (C32 and WM115) after exposure to PLX4720. Mean values of four biological replicates are shown. Protein levels represent log2 fold change of each signal (at a specific dose and time) relative to a DMSO-treated control.

### High c-Jun activity causes resistance to apoptosis in quiescent cells concomitant with incomplete pS6 suppression

Drug resistance in melanoma is often ascribed to incomplete responsiveness of tumor cells to RAF inhibitors (Lito *et al*, 2013; Shi *et al*, 2014a). In population-level measures of signaling proteins (e.g., RPPA or Western blots of pERK levels), incomplete response gives rise to partial inhibition of a signaling pathway, but at a single-cell level it usually involves cell-to-cell variability. This is particularly true in the case of apoptosis, which is an all-or-none change in cell fate (Flusberg *et al*, 2013). Our data show that *E*_max_ < 1 for viability following exposure of even the most sensitive BRAF^V600E^ cells to RAF/MEK inhibitors, implying high cell-to-cell variability. We therefore used immunofluorescence microscopy to monitor the activity of the JNK/c-Jun pathway in single cells and to relate activity to quiescence/senescence (which we did not rigorously distinguish) and apoptosis.

To score proliferation, we monitored phosphorylation of the retinoblastoma protein on Ser807/811, a modification that promotes cell cycle progression and is present during the S/G2/M phases of the cell cycle, and the levels of nuclear Ki-67, a marker of proliferation that scores negative only in quiescent cells (Buchkovich *et al*, 1989; Scholzen & Gerdes, 2000). Apoptosis was measured using the DEVD-NucView488/Hoechst 33342 assay described above. To monitor the effect of vemurafenib at the level of signaling, we measured S6 phosphorylation (Ser235/236). pS6 is a marker of TORC1 activity (Magnuson *et al*, 2012; Corcoran *et al*, 2013), a multiprotein complex controlled by signaling cascades such as MAPK, PI3K/AKT, and LKB1/AMPK (Roux *et al*, 2004; Shaw *et al*, 2004; Magnuson *et al*, 2012) involved in adaptation to vemurafenib (a point we return to below). pS6 levels have previously been proposed as a particularly effective predictor of resistance of melanoma cells to vemurafenib-induced apoptosis (Corcoran *et al*, 2013), and we found pS6^(Ser235/236)^ levels 24–48 h after drug treatment to be the best single predictor of apoptosis across cell lines, drugs, and doses. We asked whether JNK/c-Jun-mediated resistance to apoptosis was correlated with pS6 levels. Such a correlation might also reflect a role for c-Jun in controlling expression of proteins in the TORC1 pathway such as phosphoinositide-dependent kinase 1 (PDK1; an activator of AGC kinase families such as AKT and S6 kinases), PTEN, and EGFR (Johnson *et al*, 2000; Hettinger *et al*, 2007; Lopez-Bergami *et al*, 2010). We therefore measured c-Jun, p-cJun^(Ser73)^, pRb^(Ser807/811)^, Ki-67, and pS6^(Ser235/236)^ levels in various combinations in WM115, WM1552C, LOXIMVI, or COLO858 cells exposed to vemurafenib, JNK-IN-8 or the two drugs in combination and also in vemurafenib-treated cells depleted of *JUN* using siRNA.

**Figure 5:**
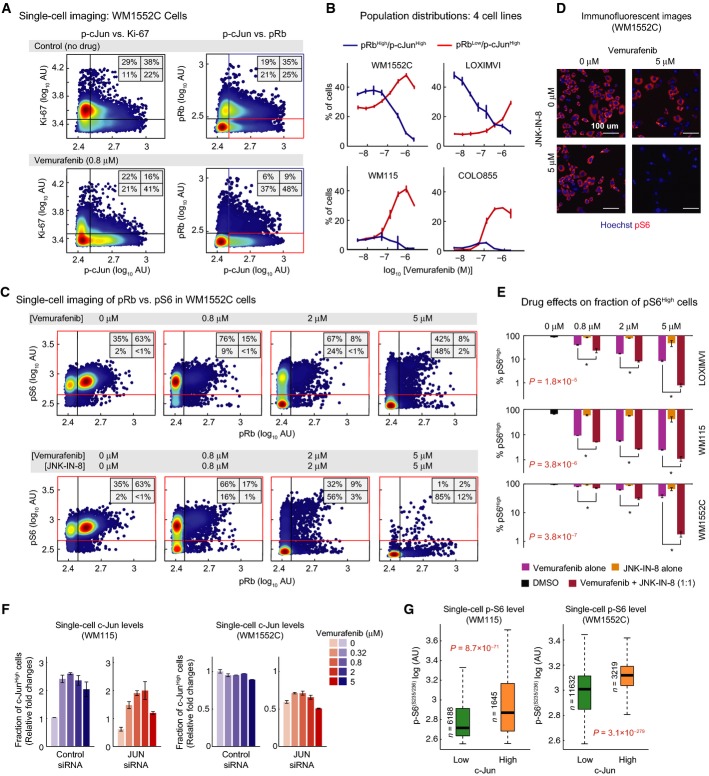
c-Jun activity up-regulation causes resistance to apoptosis in quiescent cells because of incomplete pS6 suppression

WM1552C cells were highly proliferative and largely (∽67%) Ki-67^High^ (Fig5A, top left panel; see Supplementary Fig S3A for other cell lines), but 24-h exposure to vemurafenib shifted them to a predominantly Ki-67^Low^ state (∽62% at 0.8 μM vemurafenib). The proportion of Ki-67^Low^/p-cJun^High^ cells increased concomitantly (visible as broadening of the distribution of cells along the horizontal axis of Fig5A, bottom left panel). Similar data were obtained with pRb: untreated WM1552C cells comprised ∽54% cycling pRb^High^ and ∽46% interphase pRb^low^ cells (Fig5A, top right panel; Supplementary Fig S3B). Exposure to vemurafenib reduced the proportion of pRb^High^/p-cJun^High^ cells fourfold at 0.8 μM (from ∽35% to ∽9%) and increased the proportion of pRb^Low^/p-cJun^High^ cells twofold (from ∽25% to ∽48%) (Fig5A). This shift was observed within ∽24 h of drug exposure in all four lines (Fig5B) at a time when cell killing was negligible. It thus reflects a change in the distribution of the population from proliferation to quiescence rather than death of a subset of cells. Among the four cell lines that exhibited synergistic apoptotic responses to RAF and JNK inhibitors in combination, two (WM115 and COLO858) had low basal p-cJun^High^ fractions (i.e., ∽15% and ∽3% p-cJun^High^, respectively), and vemurafenib increased the p-cJun^High^ fraction to ∽40%, a 3- to 12-fold increase, representing a clear case of JNK/c-Jun activation. In the other two lines (WM1552C and LOXIMVI), 50–60% of cells were already in a p-cJun^High^ state under normal conditions, and they retained this following exposure to vemurafenib. In all four lines, regardless of the basal p-cJun levels, vemurafenib exposure resulted in a significant increase in the proportion of quiescent p-cJun^High^ state (Fig5B). This contrasts with C32, MMACSF, and MZ7MEL cells in which p-cJun levels (and also the p-cJun^High^/pRb^Low^ subpopulation) were reduced following vemurafenib treatment (Supplementary Fig S3C). Thus, the JNK/c-Jun pathway is up-regulated or sustained in the presence of vemurafenib in about half of the lines tested, and in these cells, it is associated with a shift toward quiescence.

Covariate single-cell analysis of Ki-67 (left) and pRb^(Ser807/811)^ (right) versus p-cJun^(Ser73)^ in WM1552C cells before and 24 h after exposure to 0.8 μM vemurafenib. Density scatter plots were generated using signal intensities for individual cells as measured by immunofluorescence microscopy. The vertical lines were used to gate p-cJun^High^ versus p-cJun^Low^ cells. The horizontal lines were used to gate Ki-67^High^ versus Ki-67^Low^ cells, and pRb^H^^igh^ versus pRb^L^^ow^ cells.

Analysis of drug dose-dependent changes in proportion of pRb^L^^ow^/p-cJun^High^ and pRb^H^^igh^/p-cJun^High^ subpopulations in four melanoma cell lines (WM115, WM1552C, LOXIMVI, COLO858) after exposure to vemurafenib for 24 h. These subpopulations were gated as shown in (A). Data are represented as mean ± SD for two replicates.

Covariate single-cell analysis of pRb^(Ser807/811)^ versus pS6^(Ser235/236)^ following 24-h treatment of WM1552C cells with different doses of vemurafenib alone (top) and vemurafenib and JNK-IN-8 together (bottom). Drugs were added at a 1:1 ratio, each at indicated concentrations when used in combination. Density scatter plots were generated using signal intensities for individual cells as measured by immunofluorescence microscopy. The horizontal and vertical lines were used to gate pS6^High^ versus pS6^Low^ cells, and pRb^H^^igh^ versus pRb^L^^ow^ cells, respectively.

Selected immunofluorescence images of pS6^(Ser235/236)^ and Hoechst staining in WM1552C cells in a DMSO-treated control and 24 h after exposure to 5 μM of vemurafenib, JNK-IN-8, and their combination.

Analysis of the changes in proportion of pS6^High^ cell population in three melanoma cell lines (LOXIMVI, WM115 and WM1552C) as a function of drug concentration for single-drug vemurafenib and JNK-IN-8 treatments and their combination treatment. Drugs were added at a 1:1 ratio, each at indicated concentrations when used in combination. pS6^High^ population of cells was gated as indicated in (C). Data are represented as mean ± SD for two replicates. Data comparison between vemurafenib treatment and vemurafenib/JNK-IN-8 combined treatment was made by using two-way analysis of variance (ANOVA).

Fraction of c-Jun^High^ cells (as measured by single-cell immunofluorescence microscopy) after 48 h *JUN* knockdown followed by 24-h treatment with vemurafenib. Fold changes are shown relative to control-treated cells. Data are presented as mean ± SD.

Single-cell pS6^(Ser235/236)^ levels in the c-Jun^High^ and c-Jun^Low^ fractions of cells (as measured by single-cell multiplex immunofluorescence microscopy) after 48 h of *JUN* knockdown and 24-h treatment with 0.32 μM vemurafenib. Single-cell pS6 data are presented as box-and-whisker plots with median signal intensities and interquartile ranges; bars extending to 1.5×  the interquartile range are shown for each condition as a measure of variance. *P*-values were calculated using a two-sided nonparametric Mann–Whitney *U*-test.

Source data are available online for this figure.

To determine the consequences of co-administering vemurafenib and JNK-IN-8, we measured pS6 levels in combination with cell cycle state. In normally growing WM1552C cells, pS6^(Ser235/236)^ levels were high in both cycling pRb^High^ and interphase pRb^Low^ cells (Fig5C, far left panels). Following exposure to 0.8 μM vemurafenib for 24 h, three-quarters of cells were in interphase, but pS6 levels remained high. At 2–5 μM vemurafenib, pS6 levels began to fall, but up to 50% of the interphase pRb^Low^ cells were still pS6^High^ at 5 μM (Fig5C; top far right panel). However, when cells were exposed to vemurafenib and JNK-IN-8 together, the proportion of pS6^High^ cells fell to ∽3% (Fig5C–E) even though JNK-IN-8 alone had little effect on pS6 levels (Fig5E and Supplementary Fig S3D). A similar reduction in pS6^High^ cells was observed upon co-drugging other vemurafenib-resistant cell lines (LOXIMVI and WM115; Fig5E). We conclude that in the presence of vemurafenib, almost all cells in the population become non-proliferative, but pS6 levels remain high in a significant subset. The addition of JNK-IN-8 largely eliminates these pS6^High^ cells concomitant with an increase in apoptosis (Fig3).

When we knocked down *JUN* in WM115 and WM1552C cells by siRNA (for 48 h) and then treated cells with vemurafenib for 24 h, a significant fraction of cells died and a fraction of surviving cells appeared to remain c-Jun^High^ (due to incomplete efficiency of transfection). However, when we compared pS6 levels in c-Jun^High^ and c-Jun^Low^ survivors, we observed significantly higher levels of pS6 in the former (Fig5F and G). This further demonstrates a correlation between high pS6 and c-Jun levels among surviving cells. We hypothesize that high activity of the JNK/c-Jun pathway prevents complete pS6 inhibition and protects cells from apoptosis, partly explaining the submaximal cell killing (*E*_max_ ≪ 1) by vemurafenib. This finding is in agreement with previous data showing a strong correlation between pS6 suppression and apoptosis in BRAF^V600E^ melanoma cells exposed to RAF and MEK inhibitors.

### Estimating the magnitude of adaptive responses to RAF inhibitors and identifying biomarkers

**Figure 6:**
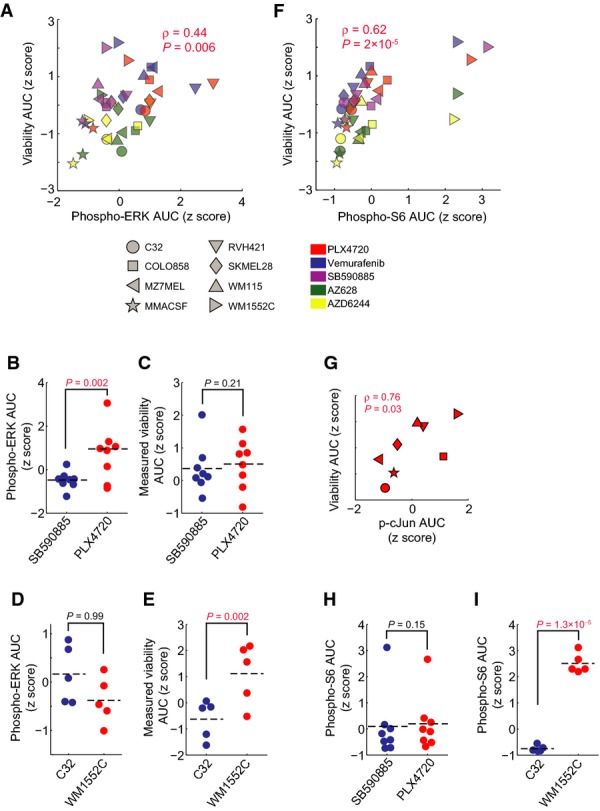
A multifactorial adaptive response, rather than initial target inhibition, determines melanoma response to RAF inhibitors

To develop an overall metric of adaptive response in RAF and MEK inhibitor-treated melanoma cells, we correlated target inhibition (as measured by pERK^(Thr202/Tyr204)^ levels) 1 h after drug exposure with viability at 72 h across different cell lines representing diverse adaptive response signatures. As expected, a statistically significant correlation was observed (*P *=* *0.006), but with scatter around the regression line (Spearman's correlation coefficient *ρ* = 0.44; Fig6A); the difference between *ρ* = 0.44 and *ρ* = 1.0 represents variability in phenotype not explained by inhibition of the primary target (leaving aside experimental error). Outliers in the regression analysis represent examples of strong and weak correlation between target inhibition and phenotypic effect. SB590885, for example, was on average significantly more effective at lowering pERK levels (*P *=* *0.002) than PLX4720 (Fig6B) but without a commensurate effect on cell viability (Fig6C). pERK fell in response to RAF/MEK inhibitors to similar degrees in C32 and WM1552C lines, but cell killing was significantly greater in the former (*P *=* *0.002; Fig6D and E). The involvement of adaptive responses in these phenomena is demonstrated by the fact that if we exclude cell lines in which p-cJun^(Ser63/73)^ up-regulation strongly attenuates vemurafenib response (COLO858, WM115, WM1552C, and LOXIMVI), the correlation between pERK levels and phenotype improves significantly (*ρ* = 0.69, *P *=* *1.4 × 10^−4^). The difference between *ρ* = 0.44 and *ρ* = 0.69 is one measure of the impact of the c-Jun-mediated adaptation to vemurafenib. More generally, the difference in the predictivity of PLSR models (that encompass multiple signaling pathways) and measures of target inhibition alone is a metric for off-pathway, adaptive, and paradoxical drug responses.

Pairwise Spearman's correlation between the 1-h changes in pERK^(Thr202/Tyr204)^ levels as measured by immunofluorescence microscopy and 72-h relative viability both represented by the *z*-score-scaled AUC of the seven dose–response curves for four RAF inhibitors and one MEK inhibitor across 8/10 cell lines investigated in this study.

Comparison of the level of 1-h pERK inhibition for 8 cell lines treated with SB590885 versus PLX4720.

Comparison of the 72-h measured relative viability AUC for 8/10 cell lines treated with SB590885 versus PLX4720.

Comparison of the level of 1-h pERK inhibition between two cell lines (C32 and WM1552C) treated with four RAF inhibitors and one MEK inhibitor.

Comparison of the 72-h measured relative viability AUC between two cell lines (C32 and WM1552C) treated with four RAF inhibitors and one MEK inhibitor.

Pairwise Spearman's correlation between the 24-h changes in pS6^(Ser235/236)^ levels as measured by immunofluorescence microscopy and 72-h relative viability both represented by the *z*-score-scaled AUC of the dose–response curves for four RAF inhibitors and one MEK inhibitor across 8/10 cell lines investigated in this study.

Pairwise Spearman's correlation between the 24-h p-cJun levels as measured by immunofluorescence microscopy and 72-h relative viability after treatment with PLX4720 for 8 cell lines. p-cJun levels are averaged over five doses (0.1–5 μM), and viability is represented by the AUC of the dose–response curves.

Comparison of the level of 24-h pS6 inhibition for 8 cell lines treated with SB590885 versus PLX4720.

Comparison of the level of 24-h pS6 inhibition between two cell lines (C32 and WM1552C) treated with four RAF inhibitors and one MEK inhibitor.

Data information: *P*-values in (B–E, H, I) were calculated using one-tailed paired Student's *t*-test. Source data are available online for this figure.

We can also use VIP scores and their correlation with phenotype to evaluate potential biomarkers of drug response (Fig2A). pS6^(Ser235/236)^ levels 24 h after treatment were the best single predictor of drug-induced cell killing in our data (*ρ* = 0.62, *P *=* *2 × 10^−6^) (Fig6F), reflecting the role of S6 kinases in integrating the activities of multiple signaling pathways. In the specific case of PLX4720, p-cJun^(Ser73)^ levels 24 h after drug exposure were also a good predictor of cell killing in 8/10 lines (*ρ* = 0.76, *P *=* *0.03) (Fig6G). We observed a significant difference in the partial correlation coefficient between viability and pS6 inhibition (controlling for pERK levels; Spearman's *ρ*_partial_ = 0.58; *P* = 1.7 × 10^−4^) and the partial correlation between viability and pERK inhibition (controlling for pS6 levels; *ρ*_partial_ = 0.34; *P* = 0.04). Thus, only a small fraction of the difference in cell killing by structurally distinct MEK and RAF inhibitors is explained by differences in the extent of target inhibition (pERK levels at 1 h) independent of pS6 changes. For example, the fact that SB590885 was no more effective than PLX4720 in killing melanoma cells despite more efficient pERK inhibition can be explained by insignificant differences in pS6 inhibition (Fig6H). Conversely, more efficient killing of C32 cells relative to WM1552C cells by PLX4720 (despite equivalent reductions in pERK) can be explained by significantly more effective suppression of pS6 (Fig6I). It has been proposed that pS6 levels better predict the responsiveness of melanoma patients to RAF inhibition than pERK levels (Corcoran *et al*, 2013), and our data are in agreement with this finding. Our data also suggest that pS6 is effective as a biomarker because it reports on the activities of pathways such as JNK/c-Jun involved in adaptive drug response. In principle, it should be possible to use regression and partial correlation in combination with mass spectrometry to identify additional and possibly better markers of drug response than pS6.

## Discussion

In this study, we apply a systematic approach to analyzing adaptive drug responses in BRAF^V600E^ melanoma, a tumor type for which adaptation to RAF and MEK inhibition has been well established. We quantify trends and variability across 10 genotypes and five drugs and identify new adaptive mechanisms. The complexity of adaptive responses challenges traditional approaches for studying signal transduction, and we therefore combined multiplex biochemical assays and single-cell phenotypic measurement with statistical modeling to identify those biochemical changes with the greatest power to predict drug-induced changes in cell viability. We find that cellular responses to RAF or MEK inhibition are remarkably diverse across cell lines—even those carrying the same BRAF^V600E^ driver—and involve multiple cell signaling kinases that can be up- or down-regulated over time, often in different directions in different cell lines. Pathways involved in adaptation extend well beyond the RAS/MEK/ERK and PI3K/AKT cascades previously shown to influence responsiveness to RAF and MEK inhibitors in melanoma cells. The plasticity of adaptive signaling, the different ways in which signaling kinases are coupled to cell state and phenotype, and the fact that we did not observe strong positive and negative correlations between different pathways raise the question of whether every cell line (or patient tumor) adapts differently to the anti-mitogenic effects of MEK or BRAF^V600E^ inhibition.

We also find that the JNK/c-Jun pathway, a primary mediator of cytokine and stress responses, is important in adaptive responses to vemurafenib. c-Jun is up-regulated in a subset of melanoma lines and co-treating cells with RAF and JNK kinase inhibitors results in a synergistic induction of apoptosis, an observation of potential therapeutic significance. The primary effect of JNK inhibition is to prevent a vemurafenib-induced shift from cycling to quiescence with a concomitant reduction in the level of apoptosis. We propose that drug-induced changes in cell cycle distribution increase cell-to-cell variability and help explain why *E*_max_ is relatively low for many MEK/RAF inhibitors even in cell lines scored as sensitive based on IC_50_. Thus, changes in the levels and activities of proteins involved in drug adaptation must be interpreted not only in light of the connectivity of the underlying pathways, but also the distribution of cell states before and after drug exposure.

### Measuring and modeling adaptive responses

In the current work, we analyzed sentinel proteins in multiple signaling cascades across time and dose for 10 cell lines. The data strongly supported this systematic design since adaptive responses altered signaling broadly and changes were often time-dependent, with the levels of some phospho-proteins falling at early times and rising at later ones and others changing monotonically. Because many samples can be assayed inexpensively in parallel, RPPA represents a good match to our experimental systems. RPPA is limited, however, to assaying proteins for which good antibodies exist and the signal-noise level is relatively poor. Future improvements to the approach include the use of alternative multiplexing methods including mass spectrometry to perform a deeper analysis of signaling under selected conditions (Fedorenko *et al*, 2015). Analysis of patient-derived cell cultures is another obvious extension, and we have previously shown that RPPA can be used to perform multiplex analysis of signaling in tumor lysates (Gujral *et al*, 2013).

Single-cell assays also proved to be important in understanding the effects of RAF inhibitors. We have found single-cell assays of apoptosis and cell number to more effectively discriminate between cytostasis and cell killing than well-average assays. We also followed up PLSR models with immunofluorescence assays as a means to correlate drug-induced changes in signaling with cell cycle state. In principle, it may be advantageous to use high-multiplicity single-cell imaging for primary data collection (Bendall *et al*, 2011; Gerdes *et al*, 2013). Live-cell imaging is also an obvious next step to determine the order and potential causality of signaling and phenotypic changes.

Partial least squares regression modeling proved remarkably effective in analyzing drug response data with models consistently predicting 90–95% of variance in response using three PLSR components (even after removing obvious cell state markers, pH3, cPARP and p27, from the analysis). The difference between this degree of predictivity and that of pERK (44% of variance explained) is a measure of the strength of adaptive responses and a figure of metric for a drug: the best drugs (or drug combinations) are those in which this difference, and thus the magnitude of adaptation, is relatively small. Another use of PLSR and related modeling methods is to evaluate potential biomarkers. pS6 has been proposed as a clinical biomarker for assessing the effectiveness of targeted therapy in BRAF^V600E^ tumors (Corcoran *et al*, 2013), and our data add the insight that c-Jun up-regulation can also be estimated by measuring pS6 levels. pS6 levels are a significantly better measure of vemurafenib-induced apoptosis than pERK inhibition (*ρ* = 0.62 versus 0.44), but neither is as good as a three-component PLSR model. This suggests that better biomarkers of vemurafenib-induced apoptosis can be identified, although these may involve multiple proteins.

By filtering signaling based on VIP scores (from PLSR models), we found that responses in melanomas are striking in their breadth (with six or more “pathways” exhibiting significant changes in activity) and diversity: the same set of kinases can rise in some cell lines and fall in others, and many responses are non-monotonic with time and dose. However, the drugs we tested had very similar effects on adaptive responses within a given cell line with the exception of known differences in mechanism, potency, and off-target binding. This is consistent with the view that adaptation is a fundamental property of a tumor cell that can be elicited by structurally distinct small molecules. However, to construct mechanistic models of these adaptive mechanisms, it will be necessary to expand the number of proteins measured and the number of perturbations introduced with siRNA or other drugs. Different network inference methods such as logical or Bayesian network modeling in combination with literature-based prior knowledge may be helpful next steps (Sachs *et al*, 2002, 2005; Morris *et al*, 2011; Saez-Rodriguez *et al*, 2011). A key question for such studies will be to determine how the diverse drug-induced changes schematized in Fig4 arise and whether they are all manifestations of a simpler phenomenon common to all cell lines.

### The JNK/c-Jun pathway as an adaptive mechanism inhibiting drug-induced apoptosis

The function of JNK in cancer is complex and context-dependent and has been linked to differential functions of the three isoforms (JNK1–JNK3) (Liu & Lin, 2005), complicating the development of JNK inhibitors as anti-cancer drugs. In some cases, inhibition of JNK signaling is clearly counter-indicated; in cutaneous squamous cell carcinoma (cSCC), the inhibition of JNK protects cells from UV-induced cell death (Vin *et al*, 2013). However, we find that the JNK/c-Jun pathway, hitherto little studied in melanoma (Lopez-Bergami *et al*, 2007), plays an important role in adaptive resistance to RAF and MEK kinase inhibitors in about half of cell lines tested. Exposing cells to vemurafenib and JNK inhibitors in combination results in synergistic cell killing. Single-cell analysis suggests that p-cJun up-regulation contributes to the resistance of a subset of the population to vemurafenib by decoupling the inhibition of proliferation from induction of apoptosis. c-Jun has well-recognized roles in regulating both proliferation and apoptosis (Wisdom *et al*, 1999), but in melanoma it is thought to function downstream of ERK by promoting transcription of cyclin D1, a positive regulator of the G1-S cell cycle transition (Lopez-Bergami *et al*, 2007). This link is evident in the first few hours after cells are exposed to RAF or MEK inhibitors when phospho-cJun levels fall dramatically. Subsequently, however, c-Jun phosphorylation is decoupled from ERK activity via processes that remain to be determined, but it is known that c-Jun activity can be elicited by growth factors, inflammatory cytokines, cAMP-dependent pathways, cellular stress, and cell cycle regulators such as Rb and cyclin-dependent kinases (de Groot & Sassone-Corsi, 1992; Nead *et al*, 1998; Wisdom *et al*, 1999; Vanden Bush & Bishop, 2011; Sun *et al*, 2014). It has also been reported that JNK activity promotes growth and survival of melanoma cells under unstressed conditions (Lopez-Bergami *et al*, 2007; Alexaki *et al*, 2008; Gurzov *et al*, 2008). Overall, our data suggest that pan-JNK inhibitors such as JNK-IN-8 are potentially useful in promoting vemurafenib-induced apoptosis in a subset of melanomas. Studies in animal models are needed to see whether a sufficient therapeutic window can be achieved to warrant further development of the concept.

### The role of fractional killing (*E*_max_ < 1) in response to RAF inhibitors

**Figure 7:**
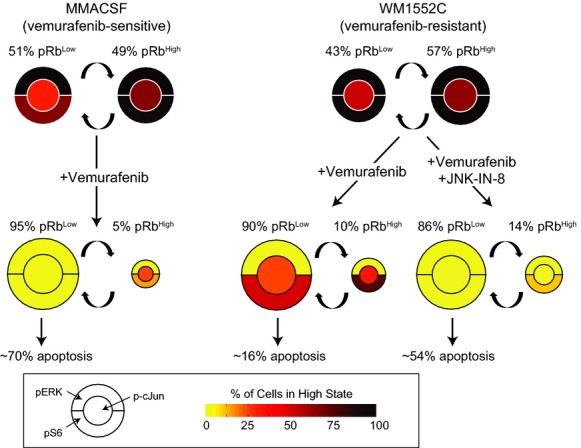
A schematic example representing c-Jun-mediated adaptive resistance to vemurafenib-induced apoptosis In the sensitive cell line (e.g., MMACSF), vemurafenib shifts the majority of the cell population toward quiescence (represented as pRb^L^^ow^ cells) and fully inhibits pERK, p-cJun, and pS6 in these cells, leading to high levels of apoptosis. In the relatively resistant cell line with p-cJun up-regulation (e.g., WM1552C), vemurafenib significantly inhibits pERK and induces quiescence in the majority of cell population, but high levels of p-cJun lead to incomplete suppression of pS6 and protect cells from apoptosis. Combination of a selective JNK inhibitor, JNK-IN-8, with vemurafenib inhibits p-cJun and pS6 in quiescent cells and increases the fraction of apoptotic cells.

Incomplete suppression of S6 phosphorylation (pS6^(Ser235/236)^) has been reported to be a good predictor of weak responsiveness to RAF inhibition in cell lines and patient-derived biopsies (Corcoran *et al*, 2013; Yuan *et al*, 2013) reflecting the role of S6 in integrating MAPK and adaptive signaling. Our studies reveal that incomplete suppression at a population level is likely to represent a bimodal response in single-cells (Fig7). In vemurafenib-resistant cell lines such as WM1552C, pS6^(Ser235/236)^ is inhibited in only ∽50% of cells even at high drug concentrations (5 μM) and remaining cells exhibit both high pS6^(Ser235/236)^ and high p-cJun levels. Virtually all cells are in interphase/quiescence under these conditions, and apoptosis appears to be inefficiently induced. We conclude that these cell-to-cell differences are one of the major causes of fractional cell killing (*E*_max_ < 1) and that JNK inhibition works by pushing cells into apoptosis, which increases maximal effect (*E*_max_). These data suggest that it will be important to use single-cell methods to study the phenotypes induced by BRAF^V600E^ inhibitors and to consider the impact of cell cycle distribution on drug IC_50_ and *E*_max_.

## Materials and Methods

### Cell lines and reagents

All melanoma cell lines were obtained from the Massachusetts General Hospital Cancer Center. C32, K2, MMACSF, SKMEL28, and WM115 cell lines were grown in DMEM/F12 (Invitrogen) supplemented with 5% fetal bovine serum (FBS) and 1% sodium pyruvate (Invitrogen). COLO858, LOXIMVI, MZ7MEL, RVH421, and WM1552C cell lines were grown in RMPI 1640 (VWR) supplemented with 5% fetal bovine serum (FBS) and 1% sodium pyruvate (Invitrogen). We added penicillin (50 U/ml) and streptomycin (50 μg/ml) to all growth media.

Chemical inhibitors from the following sources were dissolved in dimethyl sulfoxide (DMSO) as 10 mM stock solutions for *in vitro* studies: vemurafenib (PLX4032), PLX4720, SB590885, selumetinib (AZD6244) and AZ628 (all from MedChem Express), JNK-IN-8 (EMD Millipore), SP600125, doramapimod (BIRB796), and SB202190, GDC0941, tofacitinib (CP-690550), and IKK16 (all from Selleck Chemicals).

### Cell seeding and treatment

For the viability/apoptosis assays, cells were seeded at the following densities in 96-well plates (Corning) in full-growth media for 24 h: C32, MMACSF and WM115 (5,000 cells per well), MZ7MEL, RVH421, WM1552C, SKMEL28 and K2 (3,500 cells per well), and COLO858 and LOXIMVI (2,500 cells per well). Cells were then treated in 4 replicates using Hewlett-Packard (HP) D300 Digital Dispenser with either seven or nine doses (in 1:3.16 or 1:2.5 dilution ratios, respectively) of each compound for 24, 48, and 72 h. For reverse-phase protein array (RPPA) assays, all cells were plated in 4 replicates at 20,000 (or 15,000) cells per well in 96-well plates, and treated with different doses of each compound for 1, 5, 10, 24, and 48 h. Plates for RPPA assays were treated with drugs using previously prepared 384-well dilution plates and a Seiko pin transfer robot system. For 24-h immunofluorescence microscopy assays, we plated cells in two replicates, at the following densities in 96-well plates: C32, MMACSF and WM115 (10,000 cells per well), MZ7MEL, RVH421, WM1552C, SKMEL28 and K2 (7,000 cells per well), and COLO858 and LOXIMVI (5,000 cells per well).

### Cell viability and apoptosis assays

To score viability and apoptosis, we used a dye-based imaging assay; the cell-permeable DNA dye, Hoechst 33342, was used to mark nuclei and DEVD-NucView488 caspase-3 substrate was used to mark apoptosis (Tang *et al*, 2013). A total of 60 μl of a cocktail of reagents, including 4 μg/ml Hoechst 33342 (Invitrogen) and 2 μM DEVD-NucView488 caspase-3 substrate (Biotium) in phosphate-buffered saline (PBS), was dispensed into each well containing 180 μl of medium, so that the final concentrations of Hoechst 33342 and NucView488 were 1 μg/ml and 500 nM, respectively. The plates were incubated in a tissue culture incubator (37°C, 5% CO_2_) for 1.5 h. To make plate reading less time-sensitive, cells were fixed after staining, but they were not washed before imaging. A total of 26.6 μl of pre-warmed 10% paraformaldehyde in PBS was added to each well (final concentration of 1%). Plates were spun briefly at 1,000 rpm, while cells were being fixed for a total of 20 min at room temperature. Plates were then sealed using Microseal aluminum foil (Bio-Rad) and were imaged with a 10 × objective on an Operetta scanner (PerkinElmer). A total of 9 to 11 sites were imaged in each well. Image segmentation and analysis were performed using Acapella software (PerkinElmer). Nuclear segmentation using Hoechst 33342 was used to identify individual nuclei and score relative viability. To score apoptotic cells, bright spots were detected by dividing NucView488 channel nuclear intensity by the nucleus area and spots brighter than a separating threshold were scored as apoptotic. Data were analyzed using MATLAB software.

### Reverse-phase protein array (RPPA), quantitation, and analysis

We collected lysates at the designated time points after drug treatment. To generate reverse-phase arrays, lysates were printed on nitrocellulose-coated glass slides (Grace Biolabs #305177) on a 2470 Arrayer (Aushon Biosystems). Staining and analysis of RPPA data using validated antibodies were performed as previously described (Sevecka *et al*, 2011). RPPA slides were imaged initially on an Odyssey scanner (LI-COR) and subsequently on an InnoScan 710-IR scanner (Innopsys). The array images were analyzed using MicroVigene software (VigeneTech) for slides scanned on the Odyssey and Mapix software (Innopsys) for scans on the InnoScan 710-IR.

Primary antibodies used for reverse-phase protein array experiments are as follows: rabbit p-MEK^(Ser217/221)^ (Cell Signaling Technology Cat# 9154S, RRID:AB_2138017), rabbit p-ERK^(Thr202/Tyr204)^ (Cell Signaling Technology Cat# 4370, RRID:AB_2315112), rabbit p-p90RSK^(Ser380)^ (Cell Signaling Technology Cat# 9341S, RRID:AB_330753), rabbit p-p90RSK^(Thr573)^ (Cell Signaling Technology Cat# 9346S, RRID:AB_330795), rabbit p-AKT^(Thr308)^ (Cell Signaling Technology Cat# 9275L, RRID:AB_329829), rabbit p-AKT^(Ser473)^ (Cell Signaling Technology Cat# 9271L, RRID:AB_329826), rabbit p-mTOR^(Ser2448)^ (Cell Signaling Technology Cat# 2971L, RRID:AB_330971), rabbit p-p70S6K^(Thr421/Ser424)^ (Cell Signaling Technology Cat# 9204S, RRID:AB_2265916), rabbit p-p70S6K^(Thr389)^ (Cell Signaling Technology Cat# 9205S, RRID:AB_330944), rabbit p-S6^(Ser235/236)^ (Cell Signaling Technology Cat# 4858S, RRID:AB_916156), rabbit p-AMPK^(Thr172)^ (Cell Signaling Technology Cat# 2535S, RRID:AB_331250), rabbit p-JNK^(Thr183/Tyr185)^ (Cell Signaling Technology Cat# 9251L, RRID:AB_2140557), rabbit c-Jun (Cell Signaling Technology Cat# 9165, RRID:AB_2130165), rabbit p-P38^(Thr180/Tyr182)^ (Cell Signaling Technology Cat# 4511S, RRID:AB_2139682), rabbit p-HSP27^(Ser82)^ (Cell Signaling Technology Cat# 9709P, RRID:AB_11217429), rabbit p-NF-κB p65^(Ser536)^ (Cell Signaling Technology Cat# 3033L, RRID:AB_331285), rabbit c-PARP (Cell Signaling Technology Cat# 9541L, RRID:AB_331427), rabbit p-H3^(Ser10)^ (Cell Signaling Technology Cat# 3377S, RRID:AB_1549592), rabbit p27 (Cell Signaling Technology Cat# 3686S, RRID:AB_2077850), rabbit p-c-Jun^(Ser63)^ (Epitomics Cat# 1527-1, RRID:AB_562088), rabbit Bim (Epitomics Cat# 1036-1, RRID:AB_347632), and mouse β-actin antibody (Sigma Cat# A1978). Secondary antibodies are as follows: goat anti-mouse IgG conjugated to DyLight 680 (Thermo Pierce Cat# 35518, RRID:AB_614942) and goat anti-rabbit IgG conjugated to DyLight 800 (Thermo Pierce Cat# 35571, RRID:AB_614947).

RPPA data points that were out of the 1.5 × the interquartile range for the total 4 × 2 replicates (4 biological, 2 technical) were removed from the analysis. Antibodies with a Pearson correlation coefficient of <0.5 between biological replicates for each cell line were removed from the analysis. We took the median of all replicates for each condition, log2-normalized to untreated control for further analysis.

### Immunofluorescence microscopy, quantitation, and analysis

Cells were seeded and treated for the indicated times. Cells were fixed in 2% paraformaldehyde for 10 min at room temperature and washed with PBS with 0.1% Tween-20 (Sigma-Aldrich) (PBS-T), permeabilized in methanol for 10 min at room temperature, rewashed with PBS-T, and blocked in Odyssey Blocking Buffer for 1 h at room temperature. Cells were incubated overnight at 4°C with primary antibodies in Odyssey Blocking Buffer. The following primary antibodies with specified animal sources and catalog numbers were purchased and used in specified dilution ratios: rabbit p-S6^(Ser235/236)^ (Cell Signaling Technology Cat# 4858S, RRID:AB_916156), 1:800; rabbit p-ERK^(Thr202/Tyr204)^ (Cell Signaling Technology Cat# 4370, RRID:AB_2315112), 1:800; rabbit c-Jun (Cell Signaling Technology Cat# 9165, RRID:AB_2130165), 1:800; rabbit p-c-Jun^(Ser63)^ (Cell Signaling Technology Cat# 9261L, RRID:AB_2130159), 1:200; rabbit p-c-Jun^(Ser73)^ (Cell Signaling Technology Cat# 3270P, RRID:AB_2129575), 1:800; rabbit p-4EBP1^(Thr37/46)^ (Cell Signaling Technology Cat# 2855S, RRID:AB_560835), 1:200; mouse Ki-67 (Cell Signaling Technology Cat# 9449S), 1:400; mouse c-Jun (Cell Signaling Technology Cat# 2315, RRID:AB_490780), 1:200; rabbit p-AKT^(Ser473)^ (Cell Signaling Technology Cat# 4060, RRID:AB_2341228), 1:400; rabbit p-HSP27^(Ser82)^ (Cell Signaling Technology Cat# 9709P, RRID:AB_11217429), 1:800; rabbit p-STAT3^(Tyr705)^ (Cell Signaling Cat# 9145, RRID:AB_2491009), 1:200; goat p-Rb^(Ser807/Ser811)^ (Santa Cruz Biotechnology Cat# sc-16670, RRID:AB_655250), 1:400; and a mouse NF-κB p65 (Santa Cruz Biotechnology Cat# sc-8008, RRID:AB_628017), 1:400. Following treatment with primary antibodies, cells were stained with rabbit, mouse, or goat secondary antibodies labeled with Alexa Fluor 647 (Molecular Probes (Invitrogen) Cat# A31573, RRID:AB_162544), Alexa Fluor 488 (Molecular Probes (Invitrogen) Cat# A21202, RRID:AB_141607), and Alexa Fluor 568 (Molecular Probes (Invitrogen) Cat# A11057, RRID:AB_142581). Cells were washed once in PBS-T and once in PBS and were then incubated in 250 ng/ml Hoechst 33342 and 1:800 Whole Cell Stain (blue; Thermo Scientific) solutions. Cells were then washed twice with PBS and imaged with a 10 × objective on an Operetta scanner. Nine sites were imaged in each well. Image segmentation, analysis, and signal intensity quantification were performed using Acapella software. Population average and single-cell data were analyzed using MATLAB software.

### siRNA transfection

siRNA against *JUN* and a non-targeting control were from Dharmacon. WM1552C and WM115 cells were transfected using transfection reagents DharmaFECT 2 and 3 (Dharmacon), respectively, for 48 h and then treated with drugs as indicated.

### Data-driven computational modeling

We used partial least squares regression (PLSR) modeling (Geladi & Kowalski, 1986; Janes & Yaffe, 2006) to identify statistically significant covariation between molecular signals (input data; measured by RPPA) and corresponding cellular responses (output data; relative viability and apoptotic fractions) for each cell line. In our study, the dimensions of the input data matrix for each cell line were 35 × 105 (5 drugs × 7 doses; 21 signals × 5 time points). The initial dimensions for the cellular response measurements were 35 × 6 (5 drugs × 7 doses; viability and apoptotic fraction at three time points). We combined the two cellular response measurements (viability and apoptosis) at different time points to generate a new variable, “non-apoptotic viability”, by subtracting the number of apoptotic cells from the total number of cells at each condition followed by normalization to a DMSO-treated control. We then averaged the 48 and 72 h non-apoptotic viability data to generate one output variable for each of the 35 conditions in the PLSR model. The reason for this averaging is that we observe a substantial variability in the timing of responses for different cell lines exposed to different drugs. For example, both C32 and MMACSF cell lines respond with high levels of apoptosis (60–80%) to 72-h treatment with PLX4720 at doses ≥1 μM, but C32 responds more quickly (with ∽60% of apoptosis happening in the first 48 h) as compared with MMACSF (showing <20% apoptosis in the first 48 h) (see Supplementary Fig S1B). Thus, by averaging the cellular responses across the two time points, we account for the rate at which different cell lines respond to treatment (we did not use the 24 h cellular response data for PLSR modeling, as most of the cell lines do not begin to respond to treatments in the first 24 h). Accounting for averaging, the dimensions of the output data used in the PLSR models were 35 × 1. In the case of one cell line (K2), cellular response data at 72 h were unavailable and we used the 48 h data for PLSR modeling. All data were mean-centered and unit variance-scaled (*z*-score-scaled) across all conditions and time points. PLSR analysis was performed using MATLAB R2012b and “plsregress” function.

To evaluate the predictability of the linear relationship between the input and output variables in our model, we used tenfold cross-validation in which the original sample was randomly partitioned into ten subsamples. Of the ten subsamples, a single subsample was retained as the validation data for testing the model, and the remaining nine subsamples were used as training data. The cross-validation process was then repeated ten times with each of the ten subsamples used exactly once as the validation data. We computed and reported the percent of variance predicted using tenfold cross-validation. Model fitness was calculated using R^2^, Q^2^, and mean squared prediction error (MSPE) which were calculated as previously described (Gaudet *et al*, 2005). For the assessment of relative variable importance in each PLSR model, the information content of each variable (representing a signal measurement at a specific time point) was assessed by its variable importance in the projection (VIP) (Wold, 1994; Janes *et al*, 2008): 

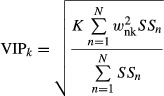
where *K* is the total number of signaling variables (*K *= 21 ×5 = 105), *w*_nk_ is the weight of the *k*^th^ variable for the *n*^th^ PLSR component, *N* is the total number of PLSR components, and *SS*_*n*_ is the sum of squares explained by the *n*^th^ PLSR component.

As described above, RPPA slides were scanned and analyzed twice using two different scanners and two different image analysis programs. The two RPPA datasets were then used independently to generate data-driven models for each cell line. The signal/time point measurements that did not show consistent up- or down-regulation between the two analyses were removed. Overall, ∽75% of the VIP data were consistent between the two analyses. Most (∽57%) of the inconsistent VIP scores (i.e., scores that had different signs between the two analyses) were insignificant (|VIP| < 1) in both analyses, and ∽37% of them were insignificant in at least one of the analyses. Only 6% of the significant VIP scores from the two analyses were not consistent. Nevertheless, we removed all of the inconsistent data from further analysis. Model-derived VIP scores for the remaining data were averaged between the two datasets and used for further analysis.

### Hierarchical clustering

Unsupervised hierarchical clustering of melanoma cell lines based on the VIP scores >1 was carried out using MATLAB and the Euclidean distance as the metric. Prior to clustering, a negative sign was added to VIP scores associated with signals that negatively correlated with viability. VIP scores of between 0 and 1 were set to zero.

### Calculating excess over Bliss independence

The Bliss independence model predicts the expected combined activity *I*_X–Y_ for two different compounds (X and Y), assuming that both single compounds act on targets interacting through independent probability events: *I*_X–Y_ = *I*_X_ + *I*_Y_ – *I*_X_.*I*_Y_, where *I*_X_ and *I*_Y_ are the single-agent activity levels at concentrations *C*_X_ and *C*_Y_. According to this model, the excess above the predicted Bliss independence represents the synergistic effect of the combination treatment (Keith *et al*, 2005).

### Statistical analyses

A one-tailed paired Student's *t*-test was used for comparing data from Fig6B–E, H, and I for which statistical significance was established for *P *<* *0.05. Data comparison between single and combined drug treatments presented in Fig5E was made by using two-way analysis of variance (ANOVA). We evaluated differences in single-cell data in Fig5G by using a nonparametric Mann–Whitney *U*-test.

### Additional online resources

All data for Supplementary Datasets S2, S3 and S4 (RPPA and viability/apoptosis measurements) and immunofluorescence microscopy experiments (including raw images) are available in a machine-readable format to facilitate re-analysis by others at http://lincs.hms.harvard.edu/db/datasets/20218/, http://lincs.hms.harvard.edu/db/datasets/20217/, and http://lincs.hms.harvard.edu/db/datasets/20219/, respectively. Further resources for exploring the data also can be found at http://lincs.hms.harvard.edu/fallahi-sichani-molsystbiol-2015/.

## Supporting Information

Supplementary Dataset S4

Review Process File
