# Supplementary material for: Systematic analysis of BRAFV600E melanomas reveals a role for JNK/c-Jun pathway in adaptive resistance to drug-induced apoptosis
Source: Mol Syst Biol. 2015 Mar 26;11(3):0797. doi: 10.15252/msb.20145877 (PMC4380931; doi:10.15252/msb.20145877)
Supplement: Supplementary file 1 [file msb0011-0797-sd1.pdf]

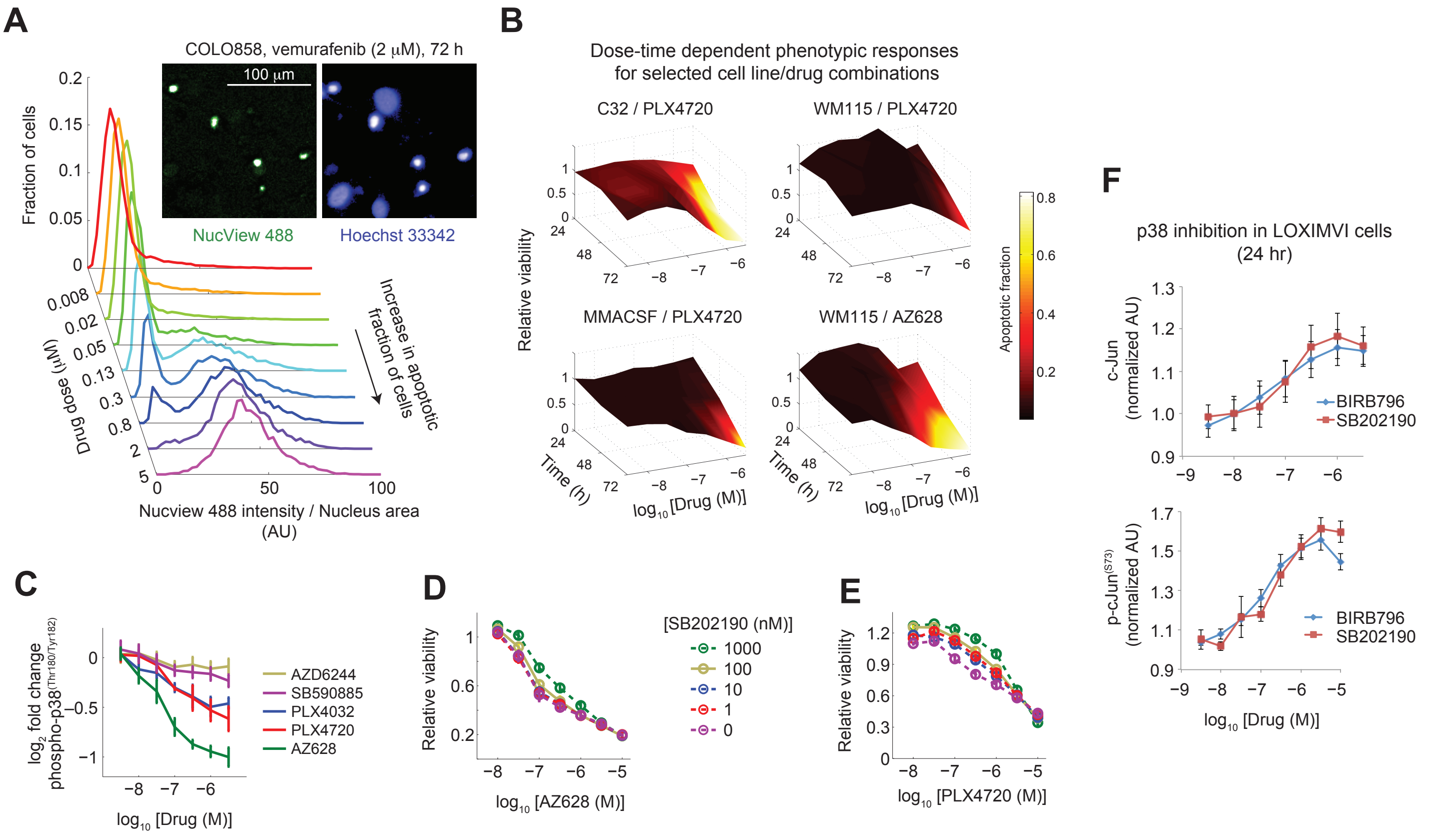

Supplementary Figure S1. Diversity in phenotypic responses of BRAF^{V600} melanoma cells to different RAF/MEK inhibitors. (A) Viability and apoptotic response measurements using a dye-based imaging assay. Cell-permeable DNA dye Hoechst 33342 was used to mark nuclei and DEVD-NucView488 caspase-3 substrate to mark apoptosis. (B) Time-dose-response measurements of relative viability and apoptotic fraction of cells for four selected cell line/drug combinations. Mean values of four biological replicates are shown. A substantial variability within the magnitude and timing of responses of different cell lines to different drugs was observed. Both C32 and MMACSF cell lines respond with high levels of apoptosis (60-80%) to 72 hr treatment with PLX4720 at doses ≥ 1 μM, but C32 responds more quickly (with ~40% of apoptosis happening in the fist 24 h) as compared with MMACSF (showing negligible apoptosis in the first 24 hr). WM115 shows a limited response to PLX4720 with negligible apoptosis. However, this cell line is more sensitive to AZ628 with higher levels of apoptosis. (C) Effect of RAF/MEK inhibition on phosphorylated p38 (Thr180/Tyr182) levels (normalized to a DMSO-treated control) as measured by RPPA. Dose-response curves for eight concentrations of five drugs are shown for LOXIMVI cells at 1 hr post-treatment. Data are presented as mean ± SD. (D,E) Effect of co-treatment of AZ628 and PLX4720 with the p38 inhibitor SB202190 on relative viability. Dose-response curves for 8 concentrations of AZ628 and PLX4720 at 5 concentrations of SB202190 (plus no SB202190) are shown for LOXIMVI cells at 72 hrs post-treatment. Data are presented as mean ± SD. (F) Effect of p38 inhibition on the mean level of total c-Jun and phosphorylated c-Jun (Ser73) per cell as measured by immunofluorescence microscopy. Data are normalized to a DMSO-treated control. Dose-response curve for 8 concentrations of SB202190 and BIRB796 are shown for LOXIMVI cells at 24 hrs post-treatment. Data are presented as mean ± SD.
