# Supplementary material for: Systematic analysis of BRAFV600E melanomas reveals a role for JNK/c-Jun pathway in adaptive resistance to drug-induced apoptosis
Source: Mol Syst Biol. 2015 Mar 26;11(3):0797. doi: 10.15252/msb.20145877 (PMC4380931; doi:10.15252/msb.20145877)
Supplement: Supplementary file 2 [file msb0011-0797-sd2.pdf]

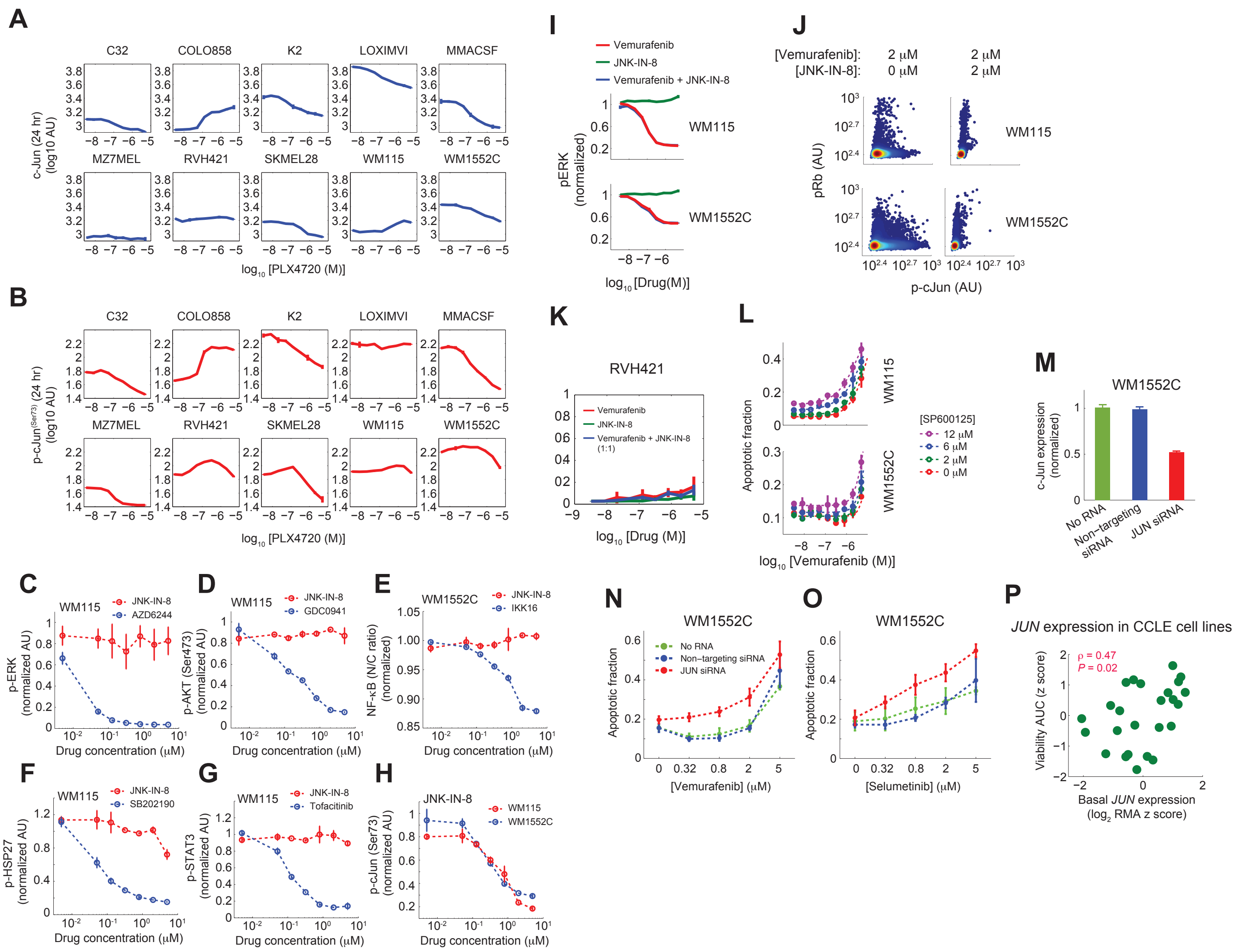

Supplementary Figure S2. High c-Jun activity up-regulation by RAF inhibitors causes resistance to apoptosis. (A,B) c-Jun and p-c-Jun$^{(Ser73)}$ changes as measured by immunofluorescence microscopy in ten melanoma cell lines in response to different doses of PLX4720 for 24 hr. Data are represented as mean ± SD. Note the differences in the pre-treatment level of c-Jun and p-cJun across different cell lines. The up-regulation is clearer in the case of cell lines with lower pre-treatment levels of c-Jun and p-cJun. (C-H) Cellular pathway profiling of JNK-IN-8. Cells were either not stimulated (C), or stimulated with 50 ng/ml EGF (D) for 10 min, IL-1α (E,F,H) for 30 min, or IL-6 (G) for 30 min following 16 hr serum starvation. Cells were treated with indicated compounds for 3 hr before cytokine treatment. The inclusion of control compounds and dose-response measurements serve to confirm the dynamic range of the assay. The control compounds include AZD6244 (a MEK inhibitor), GDC0941 (a PI3K inhibitor), IKK16 (an IκB kinase inhibitor), SB202190 (a p38 inhibitor), and tofacitinib (a JAK inhibitor). Assays were performed using immunofluorescence microscopy. We observed only on-target activity for JNK-IN-8 with no significant effect on other signaling pathways at concentrations up to 5 μM. (I,J) The effect of 24 hr JNK-IN-8 treatment on p-cJun$^{(Ser73)}$ and pERK$^{(Thr202/Tyr204)}$ levels in the presence or absence of vemurafenib. (I) Dose-response plots for pERK inhibition (population mean level) by vemurafenib, JNK-IN-8, and their combination as measured by immunofluorescence microscopy for two cell lines (WM115 and WM1552C). (J) Density scatter plots of pRb$^{(Ser807/811)}$ versus p-cJun$^{(Ser73)}$ signal for individual cells as measured by immunofluorescence microscopy for two cell lines (WM115 and WM1552C). (K) Dose-response profiles for apoptosis induction with vemurafenib and JNK-IN-8 combination for RVH421 cells. Data are represented as mean ± SD. (L) Dose-response profiles for apoptosis induction with vemurafenib and SP600125 combination. Data are represented as mean ± SD.

(M) c-Jun expression in WM1552C cells transfected with JUN siRNA relative to no RNA and non-targeting controls quantified in triplicate 48 hr after transfection. (N, O) Apoptosis in WM1552C cells with or without 48

hr JUN knockdown after 96 hr treatment with increasing doses of vemurafenib (N) and selumetinib (O). (P) Pairwise Spearman's correlation between the basal level of *JUN* mRNA expression and relative viability as represented by the AUC of the 72 hr PLX4720 dose-response curve for 25 $BRAF^{V600E}$ melanoma cell lines as reported via the Cancer Cell Line Encyclopedia (CCLE).
