# Supplementary material for: Systematic analysis of BRAFV600E melanomas reveals a role for JNK/c-Jun pathway in adaptive resistance to drug-induced apoptosis
Source: Mol Syst Biol. 2015 Mar 26;11(3):0797. doi: 10.15252/msb.20145877 (PMC4380931; doi:10.15252/msb.20145877)
Supplement: Supplementary file 3 [file msb0011-0797-sd3.pdf]

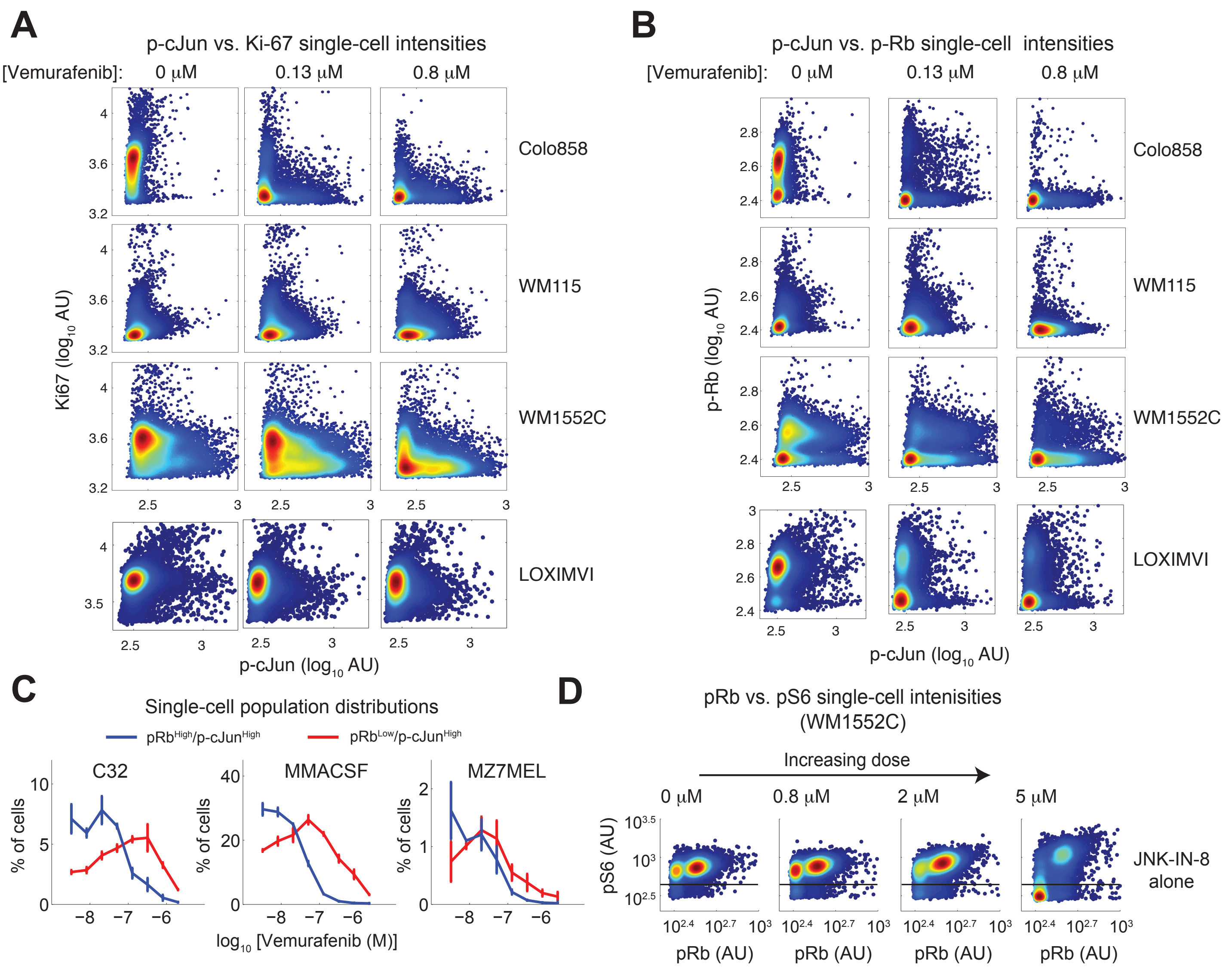

Supplementary Figure S3. High c-Jun activity causes resistance to apoptosis in quiescent cells concomitant with incomplete pS6 suppression. (A,B) Covariate single-cell analysis of Ki-67 (A) and pRb$^{(Ser807/811)}$ (B) versus p-cJun$^{(Ser73)}$ in four cell lines (COLO858, WM115, WM1552C, and LOXIMVI) before and 24 hr after exposure to 0, 0.13 and 0.8 μM vemurafenib. Density scatter plots were generated using signal intensities for individual cells as measured by immunofluorescence microscopy. (C) Analysis of drug dose-dependent changes in proportion of pRb^{Low}/p-cJun^{High} and pRb^{High}/p-cJun^{High} subpopulations in three melanoma cell lines (C32, MMACSF, and MZ7MEL) after exposure to vemurafenib for 24 hr. These subpopulations were gated as shown in Figure 5A in the main text. Data are represented as mean ± SD for two replicates. (D) Density scatter plots for pRb$^{(Ser807/811)}$ versus pS6$^{(Ser235/236)}$ individual cell signals as measured by immunofluorescent microscopy for WM1552C cells following 24 hr treatment with different doses of JNK-IN-8.
