# Supplementary material for: Systematic analysis of BRAFV600E melanomas reveals a role for JNK/c-Jun pathway in adaptive resistance to drug-induced apoptosis
Source: Mol Syst Biol. 2015 Mar 26;11(3):0797. doi: 10.15252/msb.20145877 (PMC4380931; doi:10.15252/msb.20145877)
Supplement: Supplementary file 8 [file msb0011-0797-sd8.pdf]

Systematic analysis of $BRAF^{V600E}$ melanomas reveals a role for JNK/c-Jun pathway in adaptive resistance to drug-induced apoptosis

Mohammad Fallahi-Sichani, Nathan J. Moerke, Mario Niepel, Tinghu Zhang, Nathanael Gray and Peter K. Sorger

Corresponding author: Peter K. Sorger, Harvard Medical School

Review timeline:

Submission date:	27 October 2014
Editorial Decision:	03 December 2014
Revision received:	21 January 2015
Editorial Decision:	19 February 2015
Revision received:	28 February 2015
Accepted:	04 March 2015

Editor: Maria Polychronidou

Transaction Report:

(Note: With the exception of the correction of typographical or spelling errors that could be a source of ambiguity, letters and reports are not edited. The original formatting of letters and referee reports may not be reflected in this compilation.)

1st Editorial Decision 03 December 2014

Thank you again for submitting your work to Molecular Systems Biology. We have now heard back from the three referees who agreed to evaluate your manuscript. As you will see below, the reviewers acknowledge that you address an interesting topic. However, they raise a series of concerns and make suggestions for modifications, which we would ask you to address in a revision of this work.

The concerns of the referees are rather clear and mostly refer to relatively minor technical issues. We would like however to draw you attention to the following, somewhat more substantial, points:
- Reviewer #1 refers to the need to further expand on how cJun was chosen over other candidates and on how single-cell and population-level data could be integrated.
- The reviewers suggest avoiding overstatements regarding the mechanisms mediating resistance.

Thank you for submitting this paper to Molecular Systems Biology.

Reviewer #1:

In this article, Sorger and colleagues investigate the effect of inhibiting RAF/MEK signaling in 10 different human melanoma cell lines using multiple RAF and MEK inhibitors. The authors quantified activation of host of proteins in cell population and single cells based assays at multiple time points and different inhibitor doses to quantify the cell responses. The main result of the analysis of the acquired data using a PLSR based method is the observation that the effect of the

MEK/RAF inhibitors can substantially vary between the cell lines and the characterization of the synergy between JNK and RAF/MEK signaling in affecting apoptosis/quiescence in these cells. The authors certainly address an important and interesting problem here that pertains to variability between signaling responses in different cells and finding a drug combination in this background to control tumor growth. The systems level investigation carried out by the team is commendable. However, I think, a major drawback of the approach is the lack of a tight integration between the in silico (PLSR) approach and the experiments. Therefore, the analyses produced a set of results/facts that are weakly connected with each other without creating a platform for further experimentation. This also makes the framework difficult to apply in a different biological system. I detail my comments below.

1. The PLSR analysis (Fig. 1) showed that signaling proteins (pErk, p-cJun, pS6, etc) contribute differently in each cell line in generating the responses. The authors further quantify the role of the proteins by calculating VIP scores for the proteins and selecting cJun for further study and characterizing its synergy with RAF/MEK signaling. The selection of cJun represents a crucial step as the authors are able to choose a particular candidate out of many possibilities. However, this choice appears to be quite ad hoc. How did the authors decide to choose cJun over the other candidates such as p-AMPK, pAKT, etc, which may show negative correlation between the VIP and the viability? Can the authors show that the proteins that were not chosen but had reasonable correlation between viability and VIP will not influence the RAF/MEK signaling? In the absence of such systematic approach for selecting proteins for further analysis it is not clear what is gained from this analysis over carrying out a purely experimental study and choosing few proteins for further analysis based on intuition and prior knowledge.

2. The analysis of the single cell responses showed an interesting observation that a subset of the cells treated with the drugs moved to quiescence rather than to death. In some other aspects it agreed with the previous PLSR analyses. However, this set of data remains kind of isolated from the rest of the analyses and there is no attempt to integrate the single cell and the cell population data within a predictive modeling framework. The discussion states, "..connectivity and causality inferred from single cell data looks quite different than from population level average measurements", it is unclear how this conclusion quantitatively made using the data. Perhaps, a quantitative approach, constructed to probe above statement could be starting point for creating a method for combining the population and the single cell data.

Minor comments

1. The authors state on pages 6-7, "...the C32 PLSR model captured 94% of variance in three PCs and the WM115 model captured 91% (Figure 1D; middle panels) implying that PLSR could provide meaningful insight into the connection between drug-induced signaling and phenotype." I think only handful of PCs are in general required to capture majority of the variation in reaction networks (Dworkin et al, J. R. Soc. Interf. 2012) because of the presence of strong corrections between interacting/reacting proteins. The range of the applicability of the linear relationship between the input and output variables produce should be tested using model predictions outside the training data.

2. It is not clear if the data shown on Fig. 6A and 6F show any new results than those already shown on Figs. 1-2. I think it will be helpful for the readers to follow the logic and results of the paper if fewer results are shown.

3. It will be helpful for the readers if numbers are associated with the symbols in the time and dosage legends on Fig. 1.

4. A mathematical formula for calculating the VIP score should be shown in the methods section. The definition provided in the text is not very useful in the present form.

Reviewer #2:

The manuscript by Fallahi-Sichani and colleagues describes a combined experimental-computational approach to unveil mechanisms of adaptation to drugs in which drug response of a panel of BRAF mutant melanoma cells is studied on the level of pathway activity and drug

efficiency. More precisely, the authors perturbed 10 such cell lines with 7 drugs at multiple time points and measured the response of pathways using reverse-phase protein arrays and cell viability and IC50. They then used partial least squares regression (PLSR), a method that has been previously used in similar settings, to build predictive models that link response of signaling activity to phenotypic responses. They use VIP scores to analyze which signaling nodes and time points are most relevant for reducing viability, and found that Jun/Jnk at later time points links to viability in several cell lines, and observed an induction of Jun/Jnk in several cell lines when treated with the BRAF inhibitior, suggesting that Jnk/Jun activation by the inhibitor mediates adaptation/resistance. They then investigated more in detail using additional experiments on selected cell lines if JNK inhihibition synergizes with BRAF inhibition, and present data that suggests that Jun-activation following BRAF inhibition leads to quiescent states of cells which are resistant to apoptosis.

The question of resistance against BRAF inhibitors in melanoma is very timely and interesting for the cancer research community. The paper is well written.

Overall I find most of conclusions of the paper well supported. However, I find that the connection that the authors draw to acquired resistance is purely speculative and should be removed. The authors show a mechanism of intrinsic resistance (which is very nice and very important), and it may be that intrinsic resistance of a fraction of cells could be a mechanism on which acquired resistance emerges. But the paper contains no data on that.

Apart from this, I have rather smaller technical comments and suggestions on how .

- The input and output matrices for PLS are not very well described. Why and how are multiple time points sometimes averaged? What are the exact dimensions? For the output vector, my understanding is that the dimensionality should be (number of assays = 2*3) * (number of inhibitors = 5) * (number of doses = 7). Instead it is 21*6. How was the dimensionality after averaging, i.e. which data actually entered the analysis? Maybe a figure describing the data matrices would be helpful.
- One could think that one could train a model on the combined data for all cell lines, which should be better in predicting unseen cell lines and should unveil more general and robust markers Have you tried it and how well would it predict?
- They usually get a very high performance in the PLSR. It would be interested to see how much this is attributed to the use of multiple drugs, or multiple time points. I.e. it would be very important for future papers if they took information out of the model and tested how well it performed.
- There is very little discussion on how the approach connects or compares to other approaches that are more mechanistic in nature and which could be trained by similar perturbation data (i.e. regression models, Boolean models, modular response analysis models)
- Only a fraction of cells shows the adaptive response. Can the authors speculate about the mechanism?

Minor

- It is obscuring the paper that I only learn on page 26 (materials and methods) that there are actually 2 models per cell line, which are separately analyzed and only those with consistent data enter the VIP analysis. It would be important to know how many were consistent, which have been removed etc.
- Throughout the paper they use the notation "PC components" or "number of PCs". It should rather be "PLSR comments". (see eg. http://www.sciencedirect.com/science/article/pii/S0003267006024603), as there are similarities to principle components, but it is not precisely the same.
- The definition of VIP score on p8 is not well understandable. They should also provide the reference to the Janes et al 2008 on p8.
- What are the precise thresholds used in the analysis of the data?
- Throughout the paper, the authors cite Yaffe's work on PLSR whenever referring to it. PLSR is much older then that and it would be fair to cite the original paper.

Reviewer #3:

In this manuscript Fallahi-Sichani et al. profile signaling events, at population and single-cell levels,

in melanoma cells that occur in response to small-molecule inhibitors of RAF and MEK that promote apoptosis. Through statistical analysis of this data compendium, the authors aim to identify critical nodes in signaling networks that mediate resistance in certain cell lines to these inhibitors. Because the acquisition of resistance is a common theme in the clinic, gaining insight into the mechanisms by which resistance occurs in melanoma is of critical importance.

The experimental methodology used here is a particularly powerful means by which to gain insight into resistance which sometimes may be attributable to the emergence of a pre-existing resistant clone. Thus it is important to understand how single-cells in phenotypically heterogeneous populations respond to small-molecules. In fact, such phenotypic heterogeneity may be an evolvable trait that provides robustness against unexpected changes in the cancer cell environment (such as exposure to chemical inhibitors of signaling pathways). Moreover, the statistical tools deployed here, which are similar to those use by Sorger and colleagues in the past, are very appropriate to his study given the complex and heterogeneous natures of the signaling responses.

Using these methods, the authors find that vemurafenib resistance can be attributed to elevated JNK signaling in a subset of quiescent cells. These quiescent cells are detected by elevated levels of phosphorylated S6, which thus could serve as a potential biomarker.

Overall I am quite enthusiastic about this work. The authors have used systems-level approaches to gain new insights into the development of resistance in melanoma. I think it will be of high interest to many regular readers of Molecular Systems Biology and cancer researchers who perhaps aren't familiar with the journal.

I do have two issues which I feel could be clarified before this work is accepted.

1) The first is the relationship between JNK activity and levels of total, and phosphorylated, cJUN levels. I appreciate that cJUN and phospho-cJUN levels correlate with resistance. But by why do levels of phospho-JNK not appear to correlate with resistance (as judged by VIP scores)? Potentially this could be due to a technical reason, or may have some biological explanation. I wonder if JNK is not regulating the cJUN levels/phosphorlylation directly in the presence of vemurafenib and other inhibitors, but rather there is indirect, or another route completely to cJUN regulation in these cells - especially in the presence of vemurafenib. The authors don't in fact show that JNK inhibition decease cJUN levels/phosphorylation in the presence of vemurafenib. Moreover, while the authors do show that cJUN RNAi has similar effects as JNK inhibition, they do not show that JNK-mediated resistance is in fact dependent on cJUN. Perhaps cJUN, downstream of JNK and other signaling pathways, is also regulating levels of JNK?

Thus while the authors repeatedly imply it is a JNK-JUN axis mediating resistance (i.e. JNK phosphorylating cJUN), I am not sure they have in fact provided sufficient evidence for this. Unless I missed something obvious, I feel the authors should at least discuss this point.

2) The role of S6 phosphorylation here is unclear to me. Why did the authors select this marker? What is the relationship of S6 phosphorylation with quiescence/proliferation? Is there a signaling relationship with JNK/JUN? This issue could be largely addressed through revision of the section "High
c-Jun activity causes resistance to apoptosis in quiescent cells concomitant with incomplete pS6 suppression". The transition to this section from the former seems awkward and I think may confuse some readers.

1st Revision - authors' response 21 January 2015

Reviewer #1:
In this article, Sorger and colleagues investigate the effect of inhibiting RAF/MEK signaling in 10 different human melanoma cell lines using multiple RAF and MEK inhibitors. …….The systems level investigation carried out by the team is commendable. However, I think, a major drawback of the approach is the lack of a tight integration between the in silico (PLSR) approach and the experiments. Therefore, the analyses produced a set of results/facts that are weakly

connected with each other without creating a platform for further experimentation. This also makes the framework difficult to apply in a different biological system. I detail my comments below.

In this paper, we propose a general framework based on systematic measure-model approaches to score and analyze complex adaptive changes in response to targeted therapeutics. This framework has three major stages. The first stage involves high-density dose- and time-dependent protein measurements. The main goal of these measurements is to rapidly, albeit quantitatively, screen many conditions (across multiple cell lines, drugs, drug doses, and time-points) and to measure as many relevant signals as possible. Given the large number of samples and diversity of signals (e.g. ~7,000 conditions × 21 signals in this study), it is valuable to use a multiplex, low-cost, and high-speed technology such as reverse-phase protein array (RPPA) to perform this screening. The second stage involves statistical modeling of the high-dimensional protein data, for example by constructing Partial Least Squares Regression (PLSR) models, and then analyzing data to identify those changes in signaling most predictive of response (based on the variable importance of projection – or VIP). Generated VIP scores for each cell line/signal/time-point combination can reveal what biochemical changes are most common across different cell lines and to what extent they occur on similar or different time-scales. Further, VIP measures highlight the changes that are not only common across cell lines but also phenotypically consequential. Importantly, these are not necessarily the largest fold changes, highlighting the value of PLSR-style modeling. The most significant and common VIP scores across multiple cell lines are used for the follow-up experiments in the third stage of the framework. These experiments include single-cell phenotypic and cell state assays that are planned to uncover the general importance of the identified adaptive response in resistance of cancer cells to therapy and to reveal the potential cell states that are associated with such resistance (induction of proliferation vs. induction of a quiescent, but apoptosis-resistant state). We think that this three-stage approach can be used in general for evaluating drugs with adaptive and paradoxical responses, identifying biomarkers for such responses, and suggesting potentially useful combination therapies. We have revised the manuscript to better clarify the applicability of this framework to study of other types of cancer and other therapeutic compounds.

1. The PLSR analysis (Fig. 1) showed that signaling proteins (pErk, p-cJun, pS6, etc) contribute differently in each cell line in generating the responses. The authors further quantify the role of the proteins by calculating VIP scores for the proteins and selecting cJun for further study and characterizing its synergy with RAF/MEK signaling. The selection of cJun represents a crucial step as the authors are able to choose a particular candidate out of many possibilities. However, this choice appears to be quite ad hoc. How did the authors decide to choose cJun over the other candidates such as p-AMPK, pAKT, etc, which may show negative correlation between the VIP and the viability? Can the authors show that the proteins that were not chosen but had reasonable correlation between viability and VIP will not influence the RAF/MEK signaling? In the absence of such systematic approach for selecting proteins for further analysis it is not clear what is gained from this analysis over carrying out a purely experimental study and choosing few proteins for further analysis based on intuition and prior knowledge.

As the reviewer correctly points out, we calculated VIP scores across the ten PLSR models developed for each cell line. We generated a VIP score for each three-way cell line/signal/time-point combination. Analyzing these scores (Fig. 2) shows which biochemical changes are common across different cell lines, the extent to which they occur on similar or different time-scales and whether they are phenotypically consequential. Based on this approach, we found that each cell line has a unique response and adaptive signature; c-Jun was chosen for further analysis for three reasons, any one of which we believe is sufficient to justify our choice.

- First, c-Jun mediated adaptive response is associated with one of the "most significant" VIP scores derived from the PLSR analysis in "over half of the cell lines" tested in our study as compared with other adaptive responses (AKT pathway up-regulation was another commonly observed adaptive response).

- Second, JNK/c-Jun adaptation has been very little studied as compared with those mediated by AKT and AMPK pathways (Shi et al, 2014;Yuan et al, 2013) and the analysis of JNK signaling in this context is inherently more novel analysis. It would not have been possible to identify such under-studied type of adaptation without a systematic experimental measurement followed by a rigorous analytical approach.

© European Molecular Biology Organization

- Third, c-Jun mediated adaptive response is an interesting example of a time-dependent process that involves an initial inhibition of signaling (within the first ~1-5 hr of treatment) followed by subsequent adaptation (~24-48 hr after treatment) that is well correlated with the ultimate phenotype. In all of the ten studied cell lines, the c-Jun/p-cJun levels were initially down-regulated following drug exposure and in over half of the lines they were then up-regulated. This was unexpected based on the literature and knowledge of the underlying pathways, since previous studies have reported that MEK/ERK signaling regulates c-Jun expression in $BRAF^{V600E}$ melanomas and thus, that RAF/MEK inhibitors should down-regulate c-Jun (precisely what we observed at early time points) (Lopez-Bergami et al, 2007).

- Fourth, the function of JNK/JUN pathway in cancer is of considerable general interest in the field because it is complex and context-dependent. It has been reported to have both pro- or anti-apoptotic functions, depending on cell type, nature of the death stimulus, duration of its activation and the activity of other signaling pathways (Liu & Lin, 2005). Thus, a detailed analysis in the context of adaptive drug response, and demonstrating a potential use for small molecule JNK inhibitors, is of considerable therapeutic interest.

2. The analysis of the single cell responses showed an interesting observation that a subset of the cells treated with the drugs moved to quiescence rather than to death. In some other aspects it agreed with the previous PLSR analyses. However, this set of data remains kind of isolated from the rest of the analyses and there is no attempt to integrate the single cell and the cell population data within a predictive modeling framework. The discussion states, "..connectivity and causality inferred from single cell data looks quite different than from population level average measurements", it is unclear how this conclusion quantitatively made using the data. Perhaps, a quantitative approach, constructed to probe above statement could be starting point for creating a method for combining the population and the single cell data.

We agree with the reviewer that collecting a large-scale dataset at the level of single-cell measurements might have been more informative from the outset. However, it was not clear to us how this should be performed given the number of measurements (ca 200,000) in the initial dataset and the diversity of signals we measured. Highly multiplex population average methods such as RPPA have the advantage of very low cost and high speed; we were able to measure total 21 signaling proteins and cell state markers (or their modification states) and in a single shot using RPPA. However, this comment has encouraged to revisit the question of large-scale, highly multiplex data collected for future studies.

With respect to the link between the population average and single-cell data we apologize that the reviewer was confused. The RPPA data told us which conditions lead to specific molecular changes – JNK up-regulation for example – in which cell types. They could not reveal, however, that only a subset of the cells in a population are in fact characterized by high JNK/c-Jun activity and that this negatively correlates with proliferation (as scored by pRb and Ki-67 levels). However, there is nothing in the single cell data that is discrepant with the population average data; we simply cannot visualize the cell-by-cell correlations in singles.

Overall, we believe that our data show that following up specific hypotheses made by PLSR analysis with imaging to characterize the fraction of responding and adapting cells, the fraction of cells in different cell cycle states and the correlation between the two (as depicted in Fig. 5 and Supplementary Fig. S3) is highly worthwhile. The connection between cell state (as measured by pRb and Ki-67 levels) and activity of signaling proteins (such as p-cJun and pS6) were unlikely to be uncovered. Such an approach, we note is rare in this field. We have tried to make these points more clearly in the revised manuscript.

Minor comments:

1. The authors state on pages 6-7, "...the C32 PLSR model captured 94% of variance in three PCs and the WM115 model captured 91% (Figure 1D; middle panels) implying that PLSR could provide meaningful insight into the connection between drug-induced signaling and phenotype." I think only handful of PCs are in general required to capture majority of the variation in reaction networks (Dworkin et al, J. R. Soc. Interf. 2012) because of the presence of strong

corrections between interacting/reacting proteins. The range of the applicability of the linear relationship between the input and output variables produce should be tested using model predictions outside the training data.
We agree with the reviewer that small number of PLSR components (PCs) capture the majority of the highly correlated variation in signaling changes (2-3 PCs in the case of our models). Perhaps this is not a surprising result – as implied by the reviewer – but we certainly have a case in our lab in which PLSR does not capture much variance with a few PCs. Regardless, it is an important criterion if the models are to be interpreted in terms of their VIPs.
To evaluate the predictability of the linear relationship between the input and output variables in our model, we used ten-fold cross-validation in which the original sample is randomly partitioned into ten subsamples. Of the ten subsamples, a single subsample is retained as the validation data for testing the model, and the remaining nine subsamples are used as training data. The cross-validation process is then repeated ten times with each of the ten subsamples used exactly once as the validation data. We compute and report the percent of variance predicted using ten-fold cross-validation (Q^2) for each PLSR model. The average Q^2 for the PLSR models developed for the ten studied cell lines (using three PCs) is 0.84 which shows a remarkable accuracy. It is a separate but important question whether the PCs derived from this data would also be predictive on an additional set of cell lines – this would be important in the case of biomarker development. We are starting to look into this using patient-derived melanoma lines.

2. It is not clear if the data shown on Fig. 6A and 6F show any new results than those already shown on Figs. 1-2. I think it will be helpful for the readers to follow the logic and results of the paper if fewer results are shown.
The data shown in Figure 6 are different from what is shown in Figures 1-2 with respect to the way the correlation of signaling changes – pERK or pS6 inhibition and phenotypic responses for example – are analyzed. To recap, Figures 1-2 show results derived from PLSR models developed for each individual cell line. We had observed that identifying adaptive responses is more effective if a different PLSR model is developed for each individual cell line treated (across drug type, dose and duration). This probably arises because cell lines are quite different in their responses to similar drugs, one message of our paper.
In contrast, the data presented in Figure 6 combines measurements from different cell lines. The main purpose of performing this additional analysis is: (i) to evaluate the contribution of the level of target inhibition (pERK inhibition in this case) to phenotype across different cell lines representing diverse adaptive response signatures, and (ii) to test how well measurement of proteins such as pS6 (a potential "biomarker") that report on activities of multiple pathways (including AKT/mTOR mediated adaptation (see (Corcoran et al, 2013)), and JNK/c-Jun as observed by us) can be used to predict the response. We have attempted to make this point clearer in the revised manuscript.

3. It will be helpful for the readers if numbers are associated with the symbols in the time and dosage legends on Fig. 1.
We have added the time and dosage numbers to the symbols of Figure 1.

4. A mathematical formula for calculating the VIP score should be shown in the methods section. The definition provided in the text is not very useful in the present form.
We have presented the mathematical formula used for calculating the VIP score in the Methods section of the paper.

Reviewer #2:

Overall I find most of conclusions of the paper well supported. However, I find that the connection that the authors draw to acquired resistance is purely speculative and should be removed. The authors show a mechanism of intrinsic resistance (which is very nice and very important), and it may be that intrinsic resistance of a fraction of cells could be a mechanism on which acquired resistance emerges. But the paper contains no data on that.
We are concerned that we may be working with different definitions of adaptive and intrinsic resistance. Our goal was to briefly present what we understand the cancer community to mean by short-term adaptive resistance in the development of late (acquired) resistance – citing relevant literature references. Per the reviewer's suggestion, we have revised the manuscript and removed the potentially problematic statement from the Results section of the paper and just briefly discussed the

link between short and long-term adaptive/acquired resistance in the Introduction of the manuscript with relevant citations.

Apart from this, I have rather smaller technical comments and suggestions.

- The input and output matrices for PLS are not very well described. Why and how are multiple time points sometimes averaged? What are the exact dimensions? For the output vector, my understanding is that the dimensionality should be (number of assays = 2*3) * (number of inhibitors = 5) * (number of doses = 7). Instead it is 21*6. How was the dimensionality after averaging, i.e. which data actually entered the analysis? Maybe a figure describing the data matrices would be helpful.

We thank the reviewer for catching a typo here. The initial dimensions for the cellular response measurements are 35×6 (5 drugs × 7 doses; viability and apoptotic fraction at 3 time-points). We have corrected this typo in the revised manuscript accordingly. We used the input data without any averaging in the PLSR models. However, we combined the apoptosis and viability data at each time-point to generate a new variable that we called "non-apoptotic viability". This was done by subtracting the number of apoptotic cells from the total number of cells at each condition followed by normalization to a DMSO-treated control. We then average the 48 hr and 72 hr non-apoptotic viability data to generate one output variable for each of the 35 conditions in the PLSR model (we did not include 24 hr data in averaging because most of the cell lines do not begin to respond to treatments in the first 24 hr). This does compress the time dimension and we do agree with the reviewer, and mention in the manuscript, there is no doubt more to be discovered by more explicitly analyzing time as a variable (using either the current data or new data).

The dimensionality of the final output data used in the PLSR model is 35 × 1. The main reason for this averaging is that we observe a substantial variability within the timing of responses of different cell lines to different drugs. For example, both C32 and MMACSF cell lines respond with high levels of apoptosis (60-80%) to 72 hr treatment with PLX4720 at doses ≥ 1 μM, but C32 responds more quickly (with ~60% of apoptosis happening in the fist 48 h) as compared with MMACSF (showing <20% apoptosis in the first 48 hr) (See Supplementary Table S1B). Therefore, by averaging the phenotypic responses across the two time-points, we account for the rate at which different cell lines respond to treatment.

It is helpful in this regard that we have annotated our data and provided machine-readable format data for reanalysis by others (see http://lincs.hms.harvard.edu and Supplementary Datasets 2-4). We have attempted to make these points more clearly in the revised manuscript.

- One could think that one could train a model on the combined data for all cell lines, which should be better in predicting unseen cell lines and should unveil more general and robust markers Have you tried it and how well would it predict?

We have tried it, but we found that combining all data together (at least for the ten cell lines in our study) created models that performed less well in uncovering diverse adaptive responses as compared with analysis of the cell lines individually. As alluded to above, the primary reason for this is many common adaptive responses (such as AKT and JNK/JUN pathway up-regulation) occur in both sensitive and resistant cell lines but they are not always phenotypically consequential (for undoubtedly interesting but as-yet unknown reasons). Thus, correlating the combined signaling data from all of these cell lines with viability/apoptosis data decreases predictivity, despite that greater number of data points. Nevertheless, we tried unsupervised clustering of significant VIP scores derived from the analysis of each individual cell line as depicted in Figure 2B. Interestingly, such clustering does make it possible to visualize changes in signaling associated with sensitivity to RAF/MEK inhibitors.

- They usually get a very high performance in the PLSR. It would be interested to see how much this is attributed to the use of multiple drugs, or multiple time points. i.e. it would be very important for future papers if they took information out of the model and tested how well it performed.

The reviewer makes a very good point: it is helpful to reanalyze models after removing part of the input data. We have attempted different forms of analysis and the current formulation was the easiest to interpret (as mentioned above, the data is available for re-analysis by others as well, and we will definitely be adding to it in the future).

Regarding the consequences of removing time-points, we found that most of the adaptive responses

as well as changes in cell state markers (mitosis, apoptosis and quiescence markers) occur 24-48 hr after treatment and measurements at these time-points are critical to understanding sensitivity and resistance –in contrast, we have usually been assaying the effects of drugs on cell signaling during the immediately-early period prior to 24 hr. Removing these time-points significantly reduces the performance of models. Measurements performed at earlier time-points (1-5 hr) reveal drug binding on-target (e.g. pERK inhibition) or off-target (e.g. p-p38 inhibition) and tell us about potency, but are less predictive of the phenotype than data collected at later times.

Regarding the consequences of removing drugs from the analysis, we were surprised to find this did not have a very large effect on model performance, because adaptive responses were quite similar across different compounds that target RAF or MEK (excluding known differences in drug target or poly-pharmacology). For example, the PLSR model developed form the COLO858 cell lines based on data from treatments by all five compounds has an R^2 of 0.96 and a Q^2 of 0.95. The PLSR model for the same cell line developed by use of only two of the compounds (AZD6244 and PLX4720) has an R^2 of 0.98 and a Q^2 of 0.96. To look at this another way, we removed obvious cell state markers, pH3, cPARP and p27 from the analysis (as discussed in the Discussion of the manuscript), and found that PLSR modeling still proved remarkably effective in analyzing drug response data. To our mind this is both an interesting result since it suggests that adaptive responses to MAPK dysregulation are characteristic of each cell line, but it is also a little confusing since the drugs we used are known to be very different as therapeutics. This may reflect subtle differences that were unable to detect, or different aspects of the compounds such as their pharmacokinetics.

- There is very little discussion on how the approach connects or compares to other approaches that are more mechanistic in nature and which could be trained by similar perturbation data (i.e. regression models, Boolean models, modular response analysis models)

The reviewer makes a good point. In this paper, we used PCA/PLSR based statistical modeling to identify critical determinants of response and to identify which pathways are most highly involved in drug response. We consciously selected the measurements to include a wide range of different signaling "pathways," but one consequence of this is that we do not have very detailed data on any single pathway. We strongly agree that a key step in this work is to use network inference methods such as logical modeling or Bayesian network modeling to deduce the underlying signaling biochemistry. We have added this point to the Discussion of the revised manuscript and are currently gearing up to collect the much richer data sets needed to inform such an analysis – we are hoping it can be performed on patient-derived or PDX cells to increase the translational significance.

- Only a fraction of cells shows the adaptive response. Can the authors speculate about the mechanism?

Our analysis shows that cell-to cell variability is common in adaptive responses. We and others have observed similar effects in receptor-mediated cell death (Gaudet et al, 2012;Spencer et al, 2009), activation of immune response (Feinerman et al, 2008) or sensitivity to chemotherapeutic drugs (Cohen et al, 2008) and ascribed them to stochastic fluctuation in the amounts or activities of intracellular signaling proteins. It is possible that fluctuation in target amount, activity and interaction with other proteins (Sigal et al, 2006) might also cause the adaptive response to vary from cell to cell. In the current case (in contrast to what we observed for TRAIL) cell cycle state appears to be a key variable and we speculate that it is the state of the cell at the time of initial drug response that is important. This can only be resolved by live-cell imaging however.

Minor:

- It is obscuring the paper that I only learn on page 26 (materials and methods) that there are actually 2 models per cell line, which are separately analyzed and only those with consistent data enter the VIP analysis. It would be important to know how many were consistent, which have been removed etc.

75% of the VIP data were consistent between the two analyses. It is noteworthy that most (~57%) of the inconsistent VIP scores (i.e. scores that had different signs between the two analyses) were insignificant ($|VIP| < 1$) in both analyses, and ~37% of them were insignificant in at least one of the analyses. Only 6% of the significant VIP scores from the two analyses were not consistent. Nevertheless, we removed all of the inconsistent data from further analysis. We are fairly certain this reflects noise in the data. We have added this information to the revised manuscript.

- Throughout the paper they use the notation "PC components" or "number of PCs". It should

© European Molecular Biology Organization

rather be "PLSR comments". (see e.g. http://www.sciencedirect.com/science/article/pii/S0003267006024603), as there are similarities to principle components, but it is not precisely the same.
We thank the reviewer for this revision; we have now defined PC as "PLSR component" in the revised manuscript.

- The definition of VIP score on p8 is not well understandable. They should also provide the reference to the Janes et al 2008 on p8.
This is another good suggestion; we now cite that reference on page 8 as well. In response to reviewer 1's comment, we also added the mathematical equation for calculating VIP in Methods section of the revised manuscript. We have also updated the text in an attempt to make it more intelligible.
- What are the precise thresholds used in the analysis of the data?
As mentioned in the Statistical Analysis section of the Methods, statistical significance was established for $P < 0.05$ for analyses made in Figures 5 and 6. We now make sure this information is provided in Figure legends as well.

- Throughout the paper, the authors cite Yaffe's work on PLSR whenever referring to it. PLSR is much older then that and it would be fair to cite the original paper.
This is of course true – and we apologize for the omission; we now cite a classic review on PLSR modeling: Geladi, P. & Kowalski, B. R. Partial least-squares regression — a tutorial. Anal. Chim. Acta 185, 1–17 (1986).

Reviewer #3:
Overall I am quite enthusiastic about this work. The authors have used systems-level approaches to gain new insights into the development of resistance in melanoma. I think it will be of high interest to many regular readers of Molecular Systems Biology and cancer researchers who perhaps aren't familiar with the journal.

I do have two issues which I feel could be clarified before this work is accepted.

1) The first is the relationship between JNK activity and levels of total, and phosphorylated, cJUN levels. I appreciate that cJUN and phospho-cJUN levels correlate with resistance. But by why do levels of phospho-JNK not appear to correlate with resistance (as judged by VIP scores)? Potentially this could be due to a technical reason, or may have some biological explanation. I wonder if JNK is not regulating the cJUN levels/phosphorlylation directly in the presence of vemurafenib and other inhibitors, but rather there is indirect, or another route completely to cJUN regulation in these cells - especially in the presence of vemurafenib. The authors don't in fact show that JNK inhibition decease cJUN levels/phosphorylation in the presence of vemurafenib. Moreover, while the authors do show that cJUN RNAi has similar effects as JNK inhibition, they do not show that JNK-mediated resistance is in fact dependent on cJUN. Perhaps cJUN, downstream of JNK and other signaling pathways, is also regulating levels of JNK? Thus while the authors repeatedly imply it is a JNK-JUN axis mediating resistance (i.e. JNK phosphorylating cJUN), I am not sure they have in fact provided sufficient evidence for this. Unless I missed something obvious, I feel the authors should at least discuss this point.
RPPA data show that pJNK levels also significantly increase in a subset of cell lines with c-Jun/p-cJun up-regulation (such as COLO858 and RVH421) 24-48 hr after treatment (Figure 2A). pJNK changes were not as significant in the rest of the cell lines – this may be a problem with the reagents, particularly in the case of RPPA and its problematic signal-noise level. Thus, we can only interpret the inclusion of signals in a PLSR component, not the omission. We continue to search for better means to collect multiplex protein data across large numbers of conditions.

Nevertheless, we did confirm the phosphorylation of c-Jun and its elevation following vemurafenib and that this is JNK-dependent by using JNK inhibitor JNK-IN-8 in combination with vemurafenib (see Supplementary Figure S2J). We performed the p-cJun assays at the single-cell level across four cell lines and showed that JNK inhibition synergize in cell killing with vemurafenib. Supplementary Figure S2J shows the corresponding data for WM115 and WM1552C cell lines for which other single-cell data are provided in the main figures. Thus we think that the evidence for a JNK-cJun axis is quite strong (with the obvious caveat being drug poly-pharmacology – something we now address in the paper).

It is clear, however, that we did not discuss our results sufficiently clearly or adequately marshal the arguments in favor of JNK-cJun mediated adaptation. We think that three pieces of data argue in favor of this: (i) JNK-IN-8 inhibits phosphorylation of c-Jun in a relatively specific manner (as compared with several other signaling pathways that we explicitly tested involving ERK, AKT, NF-κB, p38/HSP27, and STAT3 pathways) in the absence and presence of vemurafenib (see Supplementary Figures S2C-S2J), (ii) JNK-IN-8 enhances vemurafenib-induced apoptosis, and (iii) JUN knockdown enhances vemurafenib-induced apoptosis, provide convincing evidence that c-Jun contributes to vemurafenib resistance and this contribution is JNK-dependent.

The reviewer is absolutely correct that JNK/c-Jun pathway regulation is quite complex and involves multiple feedback loops and crosstalk with other pathways (Alexaki et al, 2008;Hui et al, 2007;Lopez-Bergami et al, 2007;Lopez-Bergami et al, 2010;Lopez-Bergami, 2011;Vanden Bush & Bishop, 2011). At this point, we cannot exclude the possibility that other pathways may have a role in c-Jun mediated adaptation and we are investigating this possibility in our future studies. We have modified the text accordingly.

2) The role of S6 phosphorylation here is unclear to me. Why did the authors select this marker? What is the relationship of S6 phosphorylation with quiescence/proliferation? Is there a signaling relationship with JNK/JUN? This issue could be largely addressed through revision of the section "High c-Jun activity causes resistance to apoptosis in quiescent cells concomitant with incomplete pS6 suppression". The transition to this section from the former seems awkward and I think may confuse some readers.

We apologize for this confusion – our interest in S6 was motivated by previous literature suggesting that S6 might be useful in the clinic as a biomarker of vemurafenib (something that seems to be holding up in initial studies) and our PLSR analysis. We found that $pS6^{(Ser235/236)}$ levels 24-48 hr after treatment were the best single predictor of cell killing in our data across different cell lines and treatments ($\rho = 0.62$, $P = 2\times10^{-6}$). The correlation for pERK (as a measure of initial target inhibition) and cell killing was significantly weaker. Studies on $BRAF^{V600E}$ melanoma cells from patients treated with vemurafenib suggest that pS6 levels better predict the responsiveness of melanoma patients to RAF inhibition than pERK levels (Corcoran et al, 2013). The same study reports that incomplete suppression of pS6 (even when pERK inhibition is complete and most cells are shifted toward quiescence as we show in our study) leads to resistance of melanoma cells to apoptosis. Further, S6 phosphorylation is an important mediator of cellular growth and metabolism, integrating signals from multiple pathways including ERK, PI3K-AKT and LKB1-AMPK pathways (Laplante & Sabatini, 2012;Ma et al, 2005;Roux et al, 2004;Shaw et al, 2004;Yuan et al, 2013). In this context, it is logical to ask whether JNK/c-Jun mediated resistance to apoptosis could be associated with pS6 levels as well. We have revised the manuscript to further clarify these points.

References

Alexaki VI, Javelaud D, Mauviel A (2008) JNK supports survival in melanoma cells by controlling cell cycle arrest and apoptosis. *Pigment Cell Melanoma Res* **21:**429-438. doi: 10.1111/j.1755-148X.2008.00466.x; 10.1111/j.1755-148X.2008.00466.x

Cohen AA, Geva-Zatorsky N, Eden E, Frenkel-Morgenstern M, Issaeva I, Sigal A, Milo R, Cohen-Saidon C, Liron Y, Kam Z, Cohen L, Danon T, Perzov N, Alon U (2008) Dynamic proteomics of individual cancer cells in response to a drug. *Science* **322:**1511-1516. doi: 10.1126/science.1160165

Corcoran RB, Rothenberg SM, Hata AN, Faber AC, Piris A, Nazarian RM, Brown RD, Godfrey JT, Winokur D, Walsh J, Mino-Kenudson M, Maheswaran S, Settleman J, Wargo JA, Flaherty KT, Haber DA, Engelman JA (2013) TORC1 suppression predicts responsiveness to RAF and MEK inhibition in BRAF-mutant melanoma. *Sci Transl Med* **5:**196ra98. doi: 10.1126/scitranslmed.3005753

Feinerman O, Veiga J, Dorfman JR, Germain RN, Altan-Bonnet G (2008) Variability and robustness in T cell activation from regulated heterogeneity in protein levels. *Science* **321:**1081-1084. doi: 10.1126/science.1158013

Gaudet S, Spencer SL, Chen WW, Sorger PK (2012) Exploring the contextual sensitivity of factors that determine cell-to-cell variability in receptor-mediated apoptosis. *PLoS Comput Biol* **8:**e1002482. doi: 10.1371/journal.pcbi.1002482

Hui L, Bakiri L, Mairhorfer A, Schweifer N, Haslinger C, Kenner L, Komnenovic V, Scheuch H,

Beug H, Wagner EF (2007) p38alpha suppresses normal and cancer cell proliferation by antagonizing the JNK-c-Jun pathway. *Nat Genet* **39:**741-749. doi: 10.1038/ng2033

Laplante M, Sabatini DM (2012) mTOR signaling in growth control and disease. *Cell* **149:**274-293. doi: 10.1016/j.cell.2012.03.017; 10.1016/j.cell.2012.03.017

Liu J, Lin A (2005) Role of JNK activation in apoptosis: a double-edged sword. *Cell Res* **15:**36-42. doi: 10.1038/sj.cr.7290262

Lopez-Bergami P (2011) The role of mitogen- and stress-activated protein kinase pathways in melanoma. *Pigment Cell Melanoma Res* **24:**902-921. doi: 10.1111/j.1755-148X.2011.00908.x; 10.1111/j.1755-148X.2011.00908.x

Lopez-Bergami P, Kim H, Dewing A, Goydos J, Aaronson S, Ronai Z (2010) c-Jun regulates phosphoinositide-dependent kinase 1 transcription: implication for Akt and protein kinase C activities and melanoma tumorigenesis. *J Biol Chem* **285:**903-913. doi: 10.1074/jbc.M109.075630; 10.1074/jbc.M109.075630

Lopez-Bergami P, Huang C, Goydos JS, Yip D, Bar-Eli M, Herlyn M, Smalley KS, Mahale A, Eroshkin A, Aaronson S, Ronai Z (2007) Rewired ERK-JNK signaling pathways in melanoma. *Cancer Cell* **11:**447-460. doi: 10.1016/j.ccr.2007.03.009

Ma L, Chen Z, Erdjument-Bromage H, Tempst P, Pandolfi PP (2005) Phosphorylation and functional inactivation of TSC2 by Erk implications for tuberous sclerosis and cancer pathogenesis. *Cell* **121:**179-193. doi: 10.1016/j.cell.2005.02.031

Roux PP, Ballif BA, Anjum R, Gygi SP, Blenis J (2004) Tumor-promoting phorbol esters and activated Ras inactivate the tuberous sclerosis tumor suppressor complex via p90 ribosomal S6 kinase. *Proc Natl Acad Sci U S A* **101:**13489-13494. doi: 10.1073/pnas.0405659101

Shaw RJ, Bardeesy N, Manning BD, Lopez L, Kosmatka M, DePinho RA, Cantley LC (2004) The LKB1 tumor suppressor negatively regulates mTOR signaling. *Cancer Cell* **6:**91-99. doi: 10.1016/j.ccr.2004.06.007

Shi H, Hong A, Kong X, Koya RC, Song C, Moriceau G, Hugo W, Yu CC, Ng C, Chodon T, Scolyer RA, Kefford RF, Ribas A, Long GV, Lo RS (2014) A novel AKT1 mutant amplifies an adaptive melanoma response to BRAF inhibition. *Cancer Discov* **4:**69-79. doi: 10.1158/2159-8290.CD-13-0279

Sigal A, Milo R, Cohen A, Geva-Zatorsky N, Klein Y, Liron Y, Rosenfeld N, Danon T, Perzov N, Alon U (2006) Variability and memory of protein levels in human cells. *Nature* **444:**643-646. doi: 10.1038/nature05316

Spencer SL, Gaudet S, Albeck JG, Burke JM, Sorger PK (2009) Non-genetic origins of cell-to-cell variability in TRAIL-induced apoptosis. *Nature* **459:**428-432. doi: 10.1038/nature08012

Vanden Bush TJ, Bishop GA (2011) CDK-mediated regulation of cell functions via c-Jun phosphorylation and AP-1 activation. *PLoS One* **6:**e19468. doi: 10.1371/journal.pone.0019468; 10.1371/journal.pone.0019468

Yuan P, Ito K, Perez-Lorenzo R, Del Guzzo C, Lee JH, Shen CH, Bosenberg MW, McMahon M, Cantley LC, Zheng B (2013) Phenformin enhances the therapeutic benefit of BRAF(V600E) inhibition in melanoma. *Proc Natl Acad Sci U S A* **110:**18226-18231. doi: 10.1073/pnas.1317577110; 10.1073/pnas.1317577110

2nd Editorial Decision 19 February 2015

Thank you again for submitting your work to Molecular Systems Biology. We have now heard back from the two referees who were asked to evaluate the study. As you will see below, the referees are satisfied with the modifications made and think that the study is now suitable for publication. While we think that the abstract modifications suggested by referee #2 are not mandatory, you are welcome to incorporate these or other changes that will allow describing the main findings more

accurately in the abstract.

Thank you for submitting this paper to Molecular Systems Biology.

Reviewer #1:

The authors have positively responded to my comments and made relevant changes to the manuscript.

Reviewer #2:

The authors addressed all my major points. I still see it problematic though to devote 30% of the abstract to aquired resistance, whereas the study focusses on adaptive response / intrinsic resistance. While it may be that this adaptive response opens a window for aquired resistance, this is still speculative and not a results of this paper. I personally find it misleading to put it in the abstract and thereby oversell the paper - which is very good as is and does not actually need overselling.

2nd Revision - authors' response 28 February 2015

Thank you again for the reviews of our manuscript entitled "Systematic analysis of BRAFV600E melanomas reveals a role for JNK/c-Jun pathway in adaptive resistance to drug-induced apoptosis" (MSB-14-5877R). In response to reviewer 2's comment, we revised the abstract and removed the sentence about acquired resistance.

© European Molecular Biology Organization
